# Histamine can be Formed and Degraded in the Human and Mouse Heart

**DOI:** 10.3389/fphar.2021.582916

**Published:** 2021-05-11

**Authors:** Joachim Neumann, Juliane M. Grobe, Jacqueline Weisgut, Hubert G. Schwelberger, Wieslawa Agnieszka Fogel, Margaréta Marušáková, Hartmut Wache, Heike Bähre, Igor B. Buchwalow, Stefan Dhein, Britt Hofmann, Uwe Kirchhefer, Ulrich Gergs

**Affiliations:** ^1^ Institut für Pharmakologie und Toxikologie, Medizinische Fakultät, Martin-Luther-Universität Halle-Wittenberg, Halle, Germany; ^2^ Department of Visceral, Transplant and Thoracic Surgery, Molecular Biology Laboratory, Medical University Innsbruck, Innsbruck, Austria; ^3^ Department of Hormone Biochemistry, Medical University of Lodz, Lodz, Poland; ^4^ Research Core Unit Metabolomics and Institute of Pharmacology, Hannover Medical School, Hannover, Germany; ^5^ Institute for Hematopathology, Hamburg, Germany; ^6^ Klinik für Herzchirurgie, Herzzentrum der Universität Leipzig, Leipzig, Germany; ^7^ Department of Cardiothoracic Surgery, Heart Centre of the University Clinics Halle (Saale), Martin-Luther-University Halle-Wittenberg, Halle, Germany; ^8^ Institut für Pharmakologie und Toxikologie, Universitätsklinikum Münster, Westfälische Wilhelms-Universität, Münster, Germany

**Keywords:** histamine synthesis and degradation, chronotropy, transgenic mice, H_2_-histamine receptor, human atrium, inotropy

## Abstract

Histamine is metabolized by several enzymes *in vitro* and *in vivo*. The relevance of this metabolism in the mammalian heart *in vivo* is unclear. However, histamine can exert positive inotropic effects (PIE) and positive chronotropic effects (PCE) in humans via H_2_-histamine receptors. In transgenic mice (H_2_-TG) that overexpress the human H_2_ receptor in cardiomyocytes but not in wild-type littermate mice (WT), histamine induced PIE and PCE in isolated left or right atrial preparations. These H_2_-TG were used to investigate the putative relevance of histamine degrading enzymes in the mammalian heart. Histidine, the precursor of histamine, increased force of contraction (FOC) in human atrial preparations. Moreover, histamine increased the phosphorylation state of phospholamban in human atrium. Here, we could detect histidine decarboxylase (HDC) and histamine itself in cardiomyocytes of mouse hearts. Moreover, our data indicate that histamine is subject to degradation in the mammalian heart. Inhibition of the histamine metabolizing enzymes diamine oxidase (DAO) and monoamine oxidase (MAO) shifted the concentration response curves for the PIE in H_2_-TG atria to the left. Moreover, activity of histamine metabolizing enzymes was present in mouse cardiac samples as well as in human atrial samples. Thus, drugs used for other indication (e.g. antidepressants) can alter histamine levels in the heart. Our results deepen our understanding of the physiological role of histamine in the mouse and human heart. Our findings might be clinically relevant because we show enzyme targets for drugs to modify the beating rate and force of the human heart.

## Introduction

**FIGURE 1:**
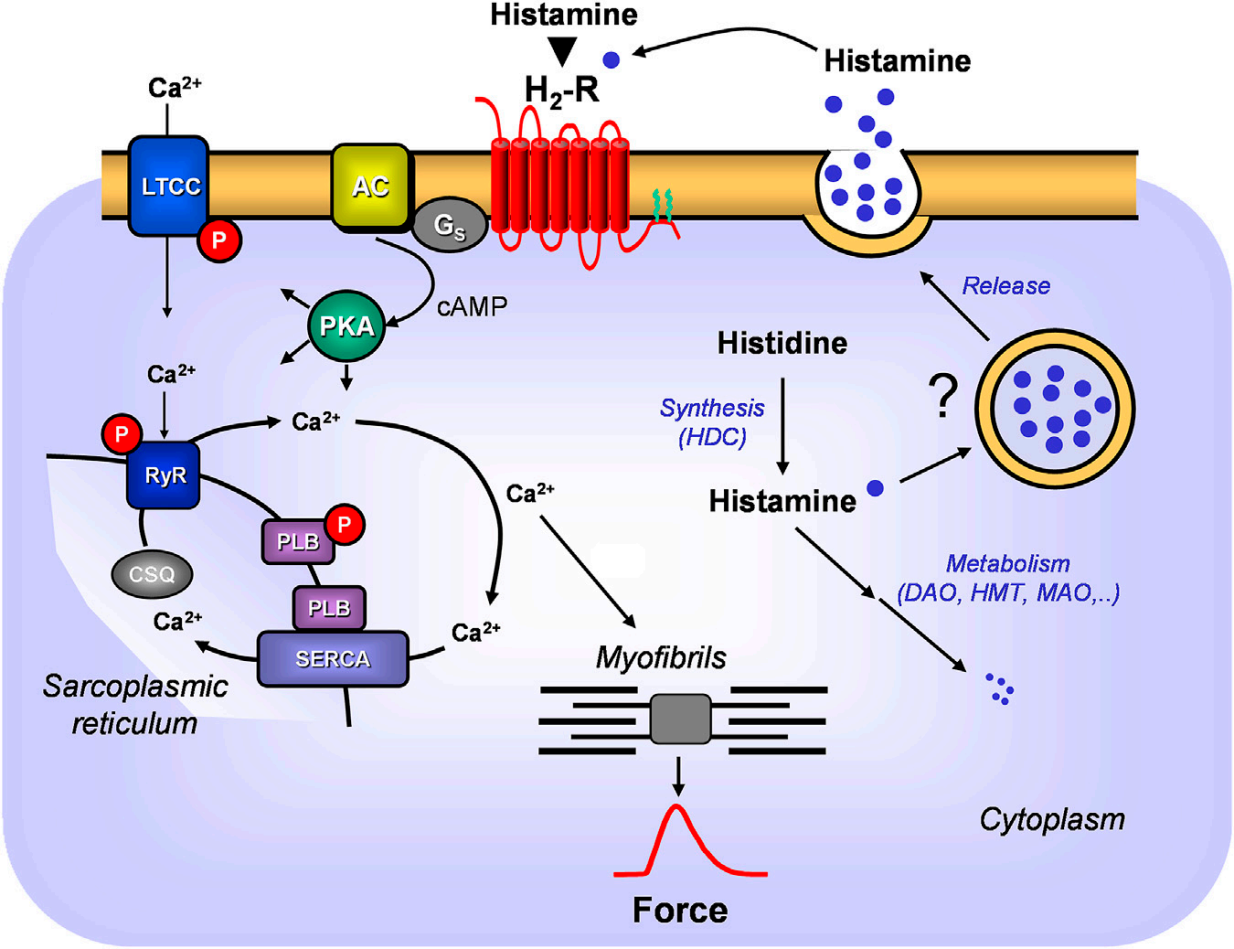
A scheme of the putative signal transduction of the H_2_-receptor in the mammalian heart. Ca^2+^ enters the mammalian heart cell via the L-type Ca^2+^ channel (LTCC). This process can be enhanced by histamine and blocked by cimetidine via a cascade starting with the H_2_-receptor. Binding of histamine elevates the activity of adenylyl cyclase (AC) in the sarcolemma via stimulatory G-proteins (Gs), augmenting thereafter the cellular cAMP and thereby stimulates the activity of cAMP-dependent protein kinase (PKA). PKA increases cardiac force generation and relaxation by increasing the phosphorylation state (P) of LTCC, phospholamban (PLB), and other regulatory proteins. Trigger Ca^2+^ initiates release of Ca^2+^ from the sarcoplasmic reticulum via ryanodine receptors (RYR) into the cytosol, where Ca^2+^ activates myofilaments and leads to increased inotropy. In diastole, Ca^2+^ is taken up into the sarcoplasmic reticulum (SR) via a sarcoplasmic reticulum Ca^2+^ ATPase (SERCA) whose activity is higher when the phosphorylation state of PLB is elevated by PKA. Ca^2+^ binds to calsequestrin (CSQ) in the SR. Moreover, the scheme depicts putative synthesis and degradation of histamine. More details are presented in Figure 2. Further, the putative storage and release of histamine is indicated.

Histamine was synthesized in a study to compare several monoamines by researchers in organic chemistry (Windaus and Vogt, 1907). Ackermann (at the same University of Freiburg, Germany) first described the conversion of histidine to histamine by bacteria (Ackermann, 1910), suggesting that histamine could be formed *in vivo* from histidine and thus could be of physiological relevance. It is now known that histamine is synthesized from histidine, for instance, in the human gut by bacteria (microbiome) but also in many organs of the human body (e.g., enterochromaffine-like cells, neurons or mast cells) and that histamine produced in such a way in the gut or ingested with food can enter the body. This is clinically relevant in some patients because they react e.g. with increased more rapid heartbeat (tachycardia) and fall in blood pressure (hypotension) to this orally applied histamine (Maintz et al., 2006; Haas et al., 2008; Schwelberger, 2010; Panula et al., 2015). The group of Sir Henry Hallet Dale in Oxford (Dale and Laidlaw, 1910) was apparently the first to publish on the positive inotropic effect (PIE, increase in force) and positive chronotropic effect (PCE, increase in heart beat) of histamine in the mammalian heart (e.g., rabbit) (Dale and Laidlaw, 1910). They also noted in rabbits a vasodilatory blood pressure reducing effect of histamine, speculated it might be involved in anaphylaxis and isolated histamine from the intestine of the rabbit, thus proving that it must be endogenously formed and were the first to show that infused histamine in animals is nearly completely metabolized (Dale and Laidlaw, 1910: isolated perfused liver). Human studies were initiated by a German gynecologist, who reported signs of allergic shock after injection of histamine including tachycardia and hypotension (Jäger, 1913a, Jäger, 1913b). Arrhythmia after injection of histamine in patients was already reported early on (Schenk, 1921) and confirmed ever after (*in vitro*, human atrium: Sanders et al., 1996). The direct cardiac effects of histamine are currently explained by stimulation of specific cardiac histamine receptors. Histamine is thought to act via only four receptors known as H_1_, H_2_, H_3_ and H_4_ histamine receptors (Jutel et al., 2009; Seifert et al., 2013; Panula et al., 2015). Regional differences in the actions of histamine or histamine receptor utilization by exogenous histamine in the mammalian heart are known: in rabbit left atrium, H_1_ receptors are more prevalent but the PIE of histamine is mediated by H_2_ receptors while in guinea pig left atrium a positive inotropic effect of histamine is mediated via H_1_ receptors (Hattori et al., 1988; Sakuma et al., 1988; Hattori et al., 1990; Hattori et al., 1991). In humans, H_1_ and H_2_ receptors were detected early on in atrium and ventricle (radioligand binding: Baumann et al., 1982; Baumann et al., 1983; Baumann et al., 1984, antibody and RNA expression: Matsuda et al., 2004). Moreover, the human cardiac H_2_ receptors were described to mediate the PIE of exogenously applied histamine in isolated human cardiac preparations (atrium: Ginsburg et al., 1980; Levi et al., 1981; Zerkowski et al., 1993; Sanders et al., 1996). These PIE in the human heart were accompanied by and hence probably mediated by an increase in cAMP (human right atrial preparations: Sanders et al., 1996) and opening of L-type Ca^2+^ channels (Eckel et al., 1982, compare Figure 1). However, phospholamban phosphorylation in the human heart in the presence of histamine was previously predicted (Sanders et al., 1996) but is now reported in the present work. Hence, the mode of action of H_2_-receptors mimics the *ß*-adrenoceptor system in the human heart.

**FIGURE 2:**
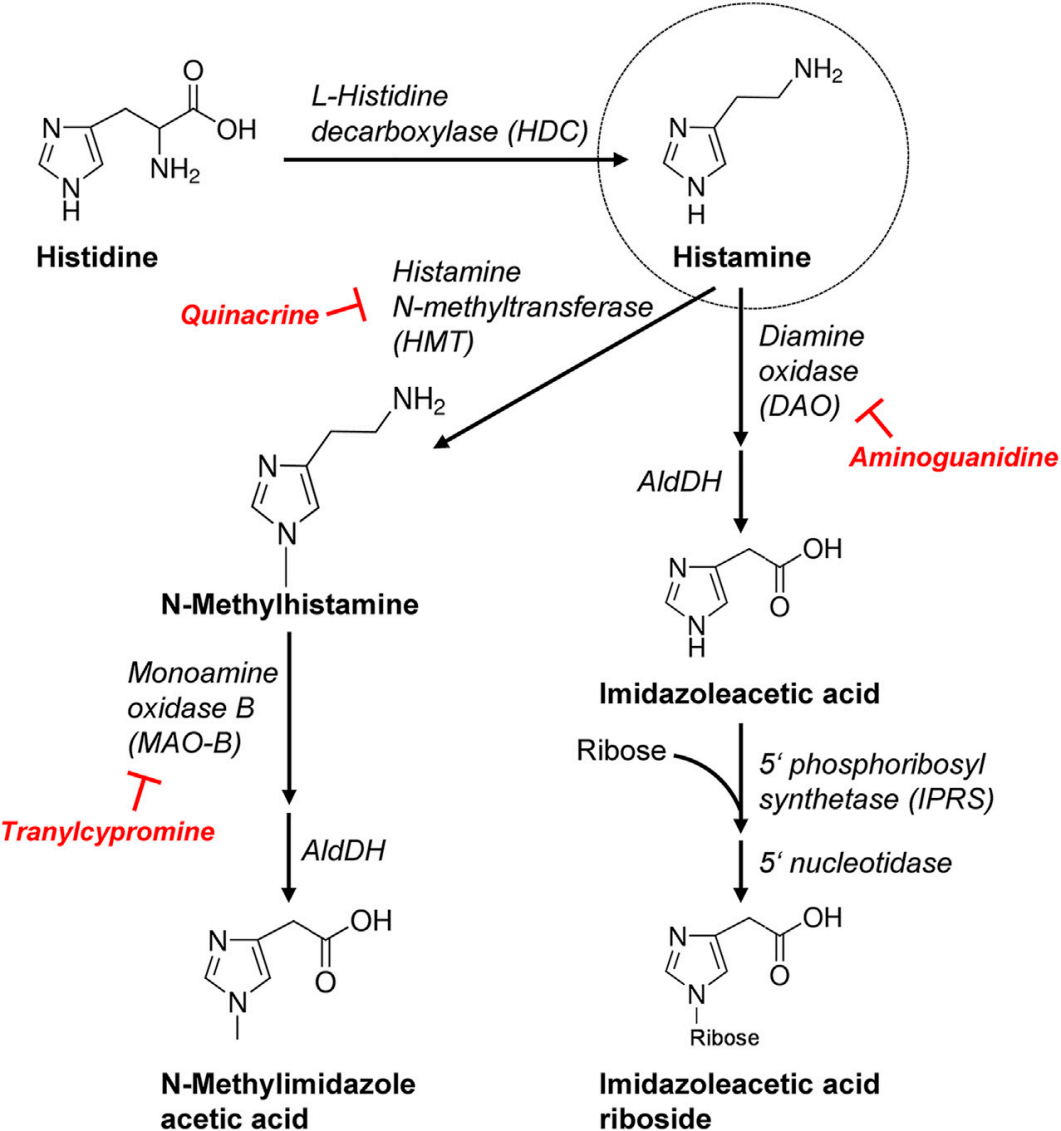
More detailed schematic presentation of histamine synthesis and degradation. Important enzymes involved are mentioned and, additionally, the inhibitors used in this study are shown. AldDH, aldehyde dehydrogenase.

Histamine is usually degraded by diamine oxidase (DAO) in the gut but higher doses of histamine can escape this pathway and can be ingested from food and thereafter histamine is delivered via blood cells to e.g. the heart (Smith and Garrett, 2005). Although peroral histamine intake is usually well tolerated, it has long been known to sometimes lead to tachycardia (humans, 500 mg; Weiss et al., 1932). A controversy exists whether histamine can also be synthesized and degraded in the heart and more specifically in cardiomyocytes, which will be addressed in the present report. We would make also the point here that histamine acts in many ways similar to serotonin (5-HT) in the heart. We reported previously that 5-HT can be formed and degraded in mouse and more importantly human hearts (Pönicke et al., 2012; Gergs et al., 2017b). Like histamine, 5-HT has no contractile effect in wild type (WT) mouse heart, but only in transgenic 5-HT_4_-overexpressing mice (5-HT_4_-TG: Gergs et al., 2013). We used this 5-HT_4_-TG to selectively monitor the contractile effect as a read out for 5-HT formation (Gergs et al., 2010; Buchwalow et al., 2013; Gergs et al., 2013). We histologically described the synthesizing and degrading enzymes in cardiomyocytes and used their inhibitors to raise or lower cardiac 5-HT levels (Gergs et al., 2017b). The same approach was used in the current communication for histamine metabolism in the heart. It was known before the start of our project that histidine via transporters can enter cells, that histidine is converted solely by histidine decarboxylase (HDC) to histamine (Figures 1, 2, for review: Haas et al., 2008; Panula et al., 2015). Moreover, HDC can be induced also in muscle cells, e. g. by stimulation with cytokines or hormones, leading to histamine synthesis followed by immediately release (Fogel, 2015). A HDC-knockout (HDC-KO) mouse has been generated and widely studied for that purpose (Ohtsu et al., 2001). Histamine can undergo two major pathways for degradation: N-methylation and oxidation via several chemical compounds until the degradation products leave the human body via the urine (Figure 2). For each of the enzymes involved, more or less specific small molecule inhibitors are available that were tested *in vitro* and *in vivo* to experimentally test these concepts (Figure 2) but also have been tried in patients for therapeutic purposes. Histamine has been detected in human hearts (ventricle: 5 μg/g: Patella et al., 1995) and was in the same order of magnitude as noradrenaline (Petch and Nayäler, 1979; Wolff and Levi, 1986), and much less in mouse hearts (0.29 μg/g: Anton and Sayre, 1969, 1–4 pmol/mg; Zimmermann et al., 2011).

The present study was started to determine whether the inotropic and chronotropic effects of histamine can be due to histamine formed and metabolized in the mouse and human heart. Preliminary reports of this project have been published in abstract form (Grobe et al., 2015; Meister et al., 2015; Grobe et al., 2016).

## Materials and Methods

### Transgenic Mice

Transgenic mice (H_2_-TG) with cardiac myocyte-specific overexpression of the human H_2_-receptor and their littermate control mice (WT) were generated as described by Gergs et al. (2019a). Heart-specific expression was achieved via the *α*-myosin heavy-chain promoter. The age of the animals ranged from three to five months. Contraction experiments were performed on left and right atrial preparations as previously described by Gergs et al. (2013). All mice were housed under conditions of optimum light, temperature, and humidity with food and water provided ad libitum. The investigation conforms to the Guide for the Care and Use of Laboratory Animals published by the National Research Council (2011). Animals were handled and maintained according to approved protocols of the animal welfare committee of the University of Halle-Wittenberg, Halle, Germany (approval reference number 42502-02-691 MLU).

### Clinical Patient Data

Right atrium samples were obtained from patients undergoing open-heart surgery with coronary artery bypass grafts, and electrically stimulated in organ baths as described previously (Gergs et al., 2013; Gergs et al., 2018; Boknik et al., 2019). Patients were treated with the following classes of drugs: ACE-inhibitors, β-adrenoceptor-blockers, diuretics, antilipemic agents, cholinergic agents, antithyroid agents, hypoglycemic agents, gout suppressants, antibiotics, α-adrenoceptor-blockers, parasympatholytics, calcium channel blockers, proton-pump inhibitors, and analgesics. Patients were in CCS scale from III to IV and NYHA class II-III. Left ventricular ejection fraction ranged from 40 to 55%. This study complied with the Declaration of Helsinki and was approved by the local ethics committee (hm-bü 04.08.2005). All patients gave informed consent.

### Contractile Studies in Mice and Men

In brief, left atrial preparations from mouse or right atrial preparations from man were isolated and mounted in organ baths as described by Neumann et al. (2003); Gergs et al. (2013); Gergs et al. (2017a); Gergs et al. (2019b). The organ baths’ bathing solution contained 119.8 mM NaCl 5.4 mM KCl, 1.8 mM CaCl_2_, 1.05 mM MgCl_2_, 0.42 mM NaH_2_PO_4_, 22.6 mM NaHCO_3_, 0.05 mM Na_2_EDTA, 0.28 mM ascorbic acid, and 5.05 mM glucose. It was continuously gassed with 95% O_2_ and 5% CO_2_ and maintained at 37°C and pH 7.4, as described by Neumann et al. (2003); Kirchhefer et al. (2004). Preparations were attached to a bipolar stimulating electrode and suspended individually in 10-ml glass tissue chambers for recording isometric contractions. Force of contraction (FOC) was measured with inductive force transducers connected to a PowerLab system (ADInstruments, Oxford, United Kingdom). Time parameters and first derivate of force of contraction of single contractions were evaluated from digitized recordings. Each muscle was stretched to the length of maximal FOC. The left atrial preparations from mice were electrically stimulated at 1 Hz with rectangular pulses of 5 m duration; the voltage was ∼10–20% greater than the threshold of initiation of contraction.

### Real Time PCR

Total RNA isolation from mouse ventricular tissue and isolated cardiac myocytes followed by reverse transcription and real time PCR were performed as described previously (Gergs et al., 2019c). Relative gene expression was calculated according to the 2^−ΔΔCt^ method (Livak and Schmittgen, 2001) with the GAPDH signal for normalization. Following primer sequences were used: HDC, forward GGA​TGA​TGG​TAC​ACT​TTG​ACT​G, reverse TGT​CTG​ACA​TGT​GCT​TGA​AGA​T (Wiener et al., 2003); GAPDH, forward ATG​CAT​CCT​GCA​CCA​CCA​AC, reverse, ATG​CCT​GCT​TCA​CCA​CCT​TC (Gergs et al., 2019c).

### Histochemical and Immunohistochemical Staining of Mouse Samples

Tissue samples of mouse hearts (or human atrial samples for MAO-A or MAO-B detection) were fixed in buffered 4% formaldehyde and routinely embedded in paraffin. PBS was used for all washings and dilutions. 4-µm-thick paraffin tissue sections were deparaffinized with xylene and graded ethanols. For pathohistological analysis, tissue sections were routinely stained with hematoxylin-eosin (HE) and Masson-Goldner-Trichrome staining (MG). For immunohistochemical assays, deparaffinaized sections were subjected to antigen retrieval by heating in 10 mM sodium citrate buffer, pH 6.0, at 95°C for 30 min.

For histamine detection, tissue samples were fixed overnight in 4% 1-ethyl-3 (3-dimethylaminopropyl)-carbodiimide (EDAC) in PBS (pH 7.4), post-fixed for 30 min in 4% formaldehyde in PBS, cryoprotected with 20% sucrose in PBS, snap-frozen in liquid nitrogen and cryosectioned at 4 μm thickness. Sections were mounted on polysine microslides (Menzel Glaeser, Braunschweig, Germany), thoroughly air-dried and stored at −20°C until use. The sections were first washed in H_2_O for 5 min and then in PBS for 10 min.

For identification of mast cells in the myocardium, Giemsa stain, as well as an enzymohistochemical reaction for visualization of specific ASD-chloroacetate-esterase activity (von Dorsche et al., 1981) were used.

Immunostaining was performed according to the standard protocol routinely used for immunohistopathology (Buchwalow and Boecker, 2010). We recently reported that endogenous Fc receptors in routinely fixed cells and tissue probes do not retain their ability to bind Fc fragments of antibodies (Buchwalow et al., 2011); therefore, blocking the endogenous Fc receptors prior to incubation with primary antibodies was omitted. After incubation with primary antibodies and following washing in PBS, sections were treated for 10 min with methanol containing 0.6% H_2_O_2_ to quench endogenous peroxidase. Bound primary antibodies were detected with the AmpliStain™ heteropolymeric HRP detection system manufactured by Stereospecific Detection Technologies (SDT GmbH, Baesweiler, Germany) (Buchwalow et al., 2013). HRP label was visualized using DAB substrate kit (Vector Laboratories, Burlingame, CA, United States). Following primary antibodies were used: rabbit polyclonal anti histidine decarboxylase (AbCam, #ab37291, dilution in PBS 1:50), rabbit polyclonal anti histamine (Chemicon/Millipore, #AB5885, 1:500 and 1:1,000), rabbit polyclonal anti monoamine oxidase A (antibodies-online, #ABIN406595, 1:200), rabbit polyclonal anti monoamine oxidase B (GeneTex, #GTX113771, 1:200), rabbit polyclonal anti histamine N-methyltransferase (abcepta, #AP1442A, 1:50 and 1:10), mouse monoclonal anti diamine oxidase (Acris/OriGene, #AM33186PU-S, 1:100 and 1:500).

Visualization and image processing. Immunostained sections were examined on a Zeiss microscope “Axio Imager Z1.” Microscopy images were captured using AxioCam digital microscope camera and AxioVision image processing (Carl Zeiss Vision, Germany). The images were acquired at 96 DPI and submitted with the final revision of the manuscript at 300 DPI. Images shown are representative of 3 independent experiments which gave similar results.

### Immunohistochemical Staining of Human Samples

For immunohistochemical staining of DAO and HMT, human heart samples were fixed for 16–24 h in 4% paraformaldehyde and embedded in paraffin wax. Sections of 5 µm were mounted on silanized glass slides (Menzel, Braunschweig, Germany), dewaxed, rehydrated, and autoclaved for 10 min at 121°C in 10 mM sodium citrate pH 6.0 for antigen retrieval. Endogenous peroxidase activity was blocked by incubation in 1% H_2_O_2_ for 15 min, endogenous biotin was blocked employing the Biotin Blocking System (Dako, Glostrup, Denmark), and non-specific protein binding sites were blocked by incubation in TNB (TBS containing 0.5% Blocking Reagent, PerkinElmer, Rodgau, Germany) for 30 min. Slides were incubated for 16 h at 4°C with the mouse monoclonal antibodies HYB313-03 specific for human DAO (Schwelberger et al., 2013) kor HYB372-07 specific for human HMT (Schwelberger et al., 2017) diluted 1:500 in TNB and then for 2 h a 25°C with horseradish peroxidase-conjugated anti-mouse immunoglobulins (Dako, Glostrup, Denmark) diluted 1:100 in TNB. The Tyramide Signal Amplification System (PerkinElmer, Rodgau, Germany) was used according to manufacturer’s instructions for signal amplification. For staining of immunocomplexes, slides were incubated for 5 min with DAB substrate (0.05% 3,3′-diaminobenzidine, 0.01% H_2_O_2_, 50 mM Tris.HCl, pH 7.6) and counterstained with Mayer's hemalum (Merck, Darmstadt, Germany). Slides were dehydrated and coverslips were mounted with Entellan (Merck, Darmstadt, Germany).

### Western Blotting of Human Samples

Human atrial tissue samples were immediately frozen and stored at −80°C until analyzed. Tissue samples (50–100 mg) were homogenized in 20 mM Tris-HCl, pH 7.0 containing 10 mM dithiothreitol and Complete Protease Inhibitor Cocktail (Roche, Vienna, Austria) for 5 min at 30 Hz using a TissueLyser II homogenizer (Qiagen, Hilden, Germany). The homogenates were cleared by centrifugation for 10 min at 20,000xg 4°C and the supernatant containing the total soluble protein was diluted in SDS sample buffer. Aliquots containing 10 µg protein were separated on 10% or 12.5% SDS polyacrylamide gels (Laemmli, 1970) and blotted onto polyvinylidene fluoride (PVDF) membranes (Towbin et al., 1979). After washing in TBST (50 mM Tris-HCl, pH 7.5, 150 mM NaCl, 0.1% Tween 20) and blocking non-specific binding sites by incubation for 60 min at 4°C in TBSTM (TBST containing 2% non-fat dry milk) the membranes were incubated for 16 h at 4°C with monoclonal antibodies HYB313-03 specific for human DAO or HYB372-07 specific for human HMT (Schwelberger et al., 2013; Schwelberger et al., 2017) diluted 1:2,000 or 1:10,000 in TBSTM, respectively. Following incubation for 60 min at 4°C with horseradish peroxidase-conjugated anti-mouse immunoglobulins (Dako, Glostrup, Denmark) diluted 1:1,500 in TBSTM, immunocomplexes were detected by incubating blots 5 min with ECL Prime reagent (GE Healthcare, Vienna, Austria) and exposure to Cronex 5 film (Agfa, Mortsel, Belgium). Protein concentration of all samples was determined by the Bradford method (Bradford, 1976) using a commercial kit (Biorad, Vienna, Austria).

### Western Blotting of Mouse Samples

For analyses of mouse enzymes, mouse cardiac homogenates were prepared in 300 µl of 10 mM NaHCO_3_ and 100 µl 20% SDS. Mixtures were kept at 25°C for 30 min before centrifugation to remove debris. Thereafter, supernatants (called homogenates) were kept at −80°C until further analysis. Western blot analysis was performed as reported (Gergs et al., 2016). Briefly, protein concentrations were determined according to the Lowry method (Lowry et al., 1951). Aliquots of 100 µg of protein were loaded per lane, transferred to nitro cellulose membranes (Towbin et al., 1979) and for immunodetection, membranes were incubated over night at 4°C with the following primary antibodies: polyclonal rabbit anti calsequestrin (#ab3516, Abcam, Cambridge, United Kingdom), monoclonal mouse anti PLB (#A010-14, Badrilla, Leeds, United Kingdom), polyclonal rabbit anti phospho-PLB (antibodies against PLB phosphorylated at serine-16 (#A010-12) or at threonine-17 (#A010-13), Badrilla, Leeds, United Kingdom), diamine oxidase (DAO, ABP1; #AM33186PU-S, Acris, Herford, Germany), monoamine oxidase A (MAO-A; #ABIN406595, antibodies-online, Aachen, Germany), monoamine oxidase B (MAO-B; #GTX113771, GeneTex (Biozol), Eching, Germany), histamine N-methyltransferase (HMT; #AP1442A, Abcepta, San Diego, CA, United States), and histidine decarboxylase (HDC; #ab37291, Abcam, Berlin, Germany). Subsequently, membranes were incubated with alkaline phosphatase-conjugated secondary antibodies (Sigma-Aldrich, Darmstadt, Germany). Bands were detected using enhanced chemifluorescence according to the manufacturer’s instructions (ECF, GE Healthcare Europe, Freiburg, Germany). Fluorescent bands were visualized in a Typhoon 9410 Variable Mode Imager (GE Healthcare Europe, Freiburg, Germany).

### Enzymes Activity Measurement

One part of the frozen tissue samples of the mouse and human cardia was analyzed with the most sensitive isotopic assays using ^14^C labeled substrates: ^14^C- serotonin for MAO A, ^14^C-beta-phenylethylamine for MAO-B, ^14^C-putrescine for DAO and ^14^C-methyl S-adenosyl-L-methionine for HMT as described elsewhere (Taylor and Snyder, 1972; Fowler and Tipton, 1982; Fogel et al., 1985). In more details, outer mitochondrial membrane bound MAO-A and MAO-B activities were estimated in the tissue homogenates using serotonin (final concentration 200 μM) and ^14^C-beta-phenylethylamine (final concentration 20 μM), as well as specific inhibitors, i.e. clorgyline and deprenyl (0.3 μM each), respectively (Fowler and Tipton, 1982). Diamine oxidase, a soluble enzyme, was assayed in supernatants with ^14^C-putrescine (300 μM) as substrate. Products of the oxidative deamination were separated by column chromatography on Dowex 50WX8 and measured by liquid scintillation counting (Fogel et al., 1985). Histamine N-methyltransferase activity (HMT) likewise was determined in the tissue supernatants using histamine as a substrate and adenosyl-L-methionine, S-[methyl-^14^C] (Perkin-Elmer) as a donor of methyl group (100 μM each) with aminoguanidine and pargyline serving as inhibitors of DAO and MAO enzymes, after Taylor and Snyder (1972). The enzyme activities were expressed as pmol/min/mg protein. Protein concentrations were analyzed according to Lowry’s method (1951).

### Mass Spectrometry

The amount of histamine in cardiac preparations (heart tissue, ventricular myocytes and plasma/serum) was quantified as follows:

The tissue was weighed and subsequently homogenized using a FastPrep 24 device (MP Biomedicals). Therefore tissue was transferred into 2 ml screw caps filled with Lysing Matrix A (MP Biomedicals). 800 µl ethanol/water (70/30; v/v) was added and the samples were homogenized two times for 30 s with 5 m/s. After centrifugation 600 µl of supernatant was transferred to a reaction tube and evaporated to dryness under a gentle nitrogen stream at 40°C. The residual pellet was reconstituted in 50 µl 80/20 acetonitrile/water (v/v) containing 0.25 µM of d4-histamine (C/D/N isotopes, Pointe-Caire, Canada) as internal standard. This solution was transferred to an analysis vial for masspectrometric analysis. Cardiac myocyte suspension was treated with a 4 fold amount of 50/50 acetonitrile/water (v/v). After centrifugation supernatant was transferred to a reaction tube and prepared for analysis as described above. For analyte extraction of murine blood or serum, 50 µl sample was treated with 200 µl of 50/50 acetonitrile/water (v/v). After centrifugation supernatant was transferred to a reaction tube and prepared for analysis as described above. The calibrators (ranging from 0.0079 to 4 pmol/sample) were prepared in 50 mg/ml BSA and aliquoted to 50 µL. The calibrators were prepared in the same way as blood/serum samples. Histamine was quantified using a liquid chromatography system coupled to a mass spectrometer (API2000; Sciex, Foster City, CA) as described previously (Zimmermann et al., 2011).

### Data Analysis

Data shown are means ± SEM. Statistical significance was estimated by analysis of variance followed by Bonferroni’s *t*-test or by student’s t-test where appropriate. A *p*-value of less than 0.05 was considered significant. Experimental data for agonist-induced positive inotropic and chronotropic effects were analyzed by fitting sigmoidal curves to the experimental data with GraphPad Prism 5.0. All other statistical analyses were performed as indicated in the figures and tables. Statistical evaluation was conducted with GraphPad Prism 5.0 (GraphPad Software, San Diego, California, United States).

### Drugs and Materials

Histidine, histamine, cimetidine, quinacrine, tranylcypromine, aminoguanidine, and compound 48/80 were purchased from Sigma-Aldrich (Taufkirchen, Germany). All other chemicals were of the highest purity grade commercially available. Deionized water was used throughout the experiments. Stock solutions were freshly prepared daily.

## Results

### Effects of Histidine and Histamine

**FIGURE 3:**
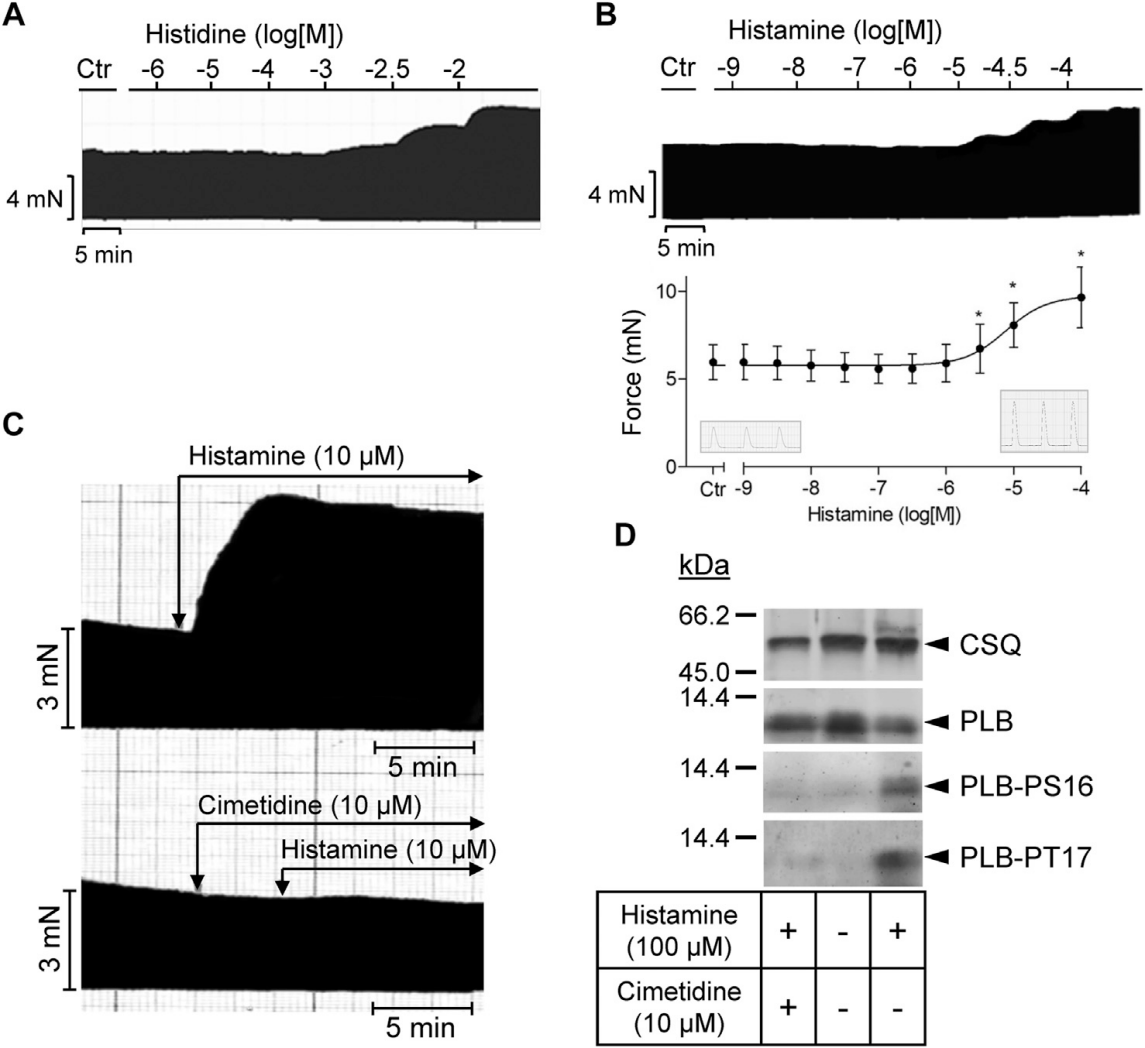
Human right atrial preparations. **(A)** Representative original recording of the concentration- and time-dependent effect of histidine on the force of contraction (FOC) in isolated electrically driven (1 Hz) atrial preparations from human heart. For comparison, the effect of histidine on the FOC in isolated electrically driven left atrial preparation from H_2_-TG mouse heart is shown as Supplementary Figure S1. **(B)** Representative original recording and quantification (n = 9) of the concentration- and time-dependent effect of histamine on the FOC in isolated electrically driven (1 Hz) atrial preparations from human heart. *p < 0.05 vs. Ctr. **(C)** Effect of 10 µM histamine alone or 10 µM histamine in the presence of 10 µM cimetidine on FOC in isolated electrically driven (1 Hz) atrial preparations from human heart: original recordings. **(D)** Representative original Western blots indicating an increase of phospholamban (PLB) phosphorylation at serine 16 (PS16) and at threonine 17 (PT17) under histamine. The PLB phosphorylation was cimetidine-sensitive. Calsequestrin (CSQ) expression was used as loading control. Molecular weight markers in kilo Dalton (kDa) are indicated. Samples used were frozen from contraction studies like those in **(C)**.

As seen in the original recording (Figure 3A), histidine exerted a PIE in isolated electrically stimulated (1 Hz) human right atrial preparations: 10 mM histidine increased force of contraction by 27% from 6.02 ± 0.63 mN to 7.66 ± 0.43 to mN (*p* < 0.05, n = 3). In the presence of NSD-1055 (100 µM), a decarboxylase inhibitor, histidine failed to exert a PIE in human atrial preparations (data not shown). For comparison, the effects of histidine (cumulatively applied) and histamine (10 µM) on a H_2_-TG mouse left atrium are shown in Supplementary Figure S1. In mouse atrial preparations, histidine was less effective than in human atrium. Similarly, histamine exerted a concentration-dependent PIE in human right atrial preparations (original recording, Figure 3B) when histamine was cumulatively applied. In a different set of patient samples, a PIE to single additions of histamine (1, 10, or 100 µM) was recorded which could be blocked by 10 µM cimetidine (Figure 3C). Histamine was more potent than histidine to elicit a PIE. Moreover, as a new finding, histamine in the set of patient samples where it was not cumulatively applied increased the phosphorylation state of PLB at serine 16 (Figure 3D), which was likewise blocked by cimetidine. This PIE of histamine at human atrium was also accompanied by shortened relaxation time and elevated maximum rate of relaxation, consistent with the known functional consequences of phospholamban phosphorylation (Table 1).

**TABLE 1:** Contractile parameters (time to peak tension (TP), time of relaxation (RT), first maximum positive or negative derivate of force development (dF/dt max or min)) in human right atrial preparations. Data are means ± SD from 8 preparations of 3 patients.

Parameters	Ctr	Histamine (100 µM)
TP (ms)	54.2 ± 2.8	43.9 ± 2.2^*^
RT (ms)	124.0 ± 11.3	108.3 ± 8.9^*^
dF/dtmax (mN/s)	71.2 ± 24.1	337.7 ± 73.4^*^
dF/dtmin (mN/s)	−40.4 ± 15.0	−183.9 ± 63.0^*^

*p < 0.05 vs. Ctr.

**FIGURE 4:**
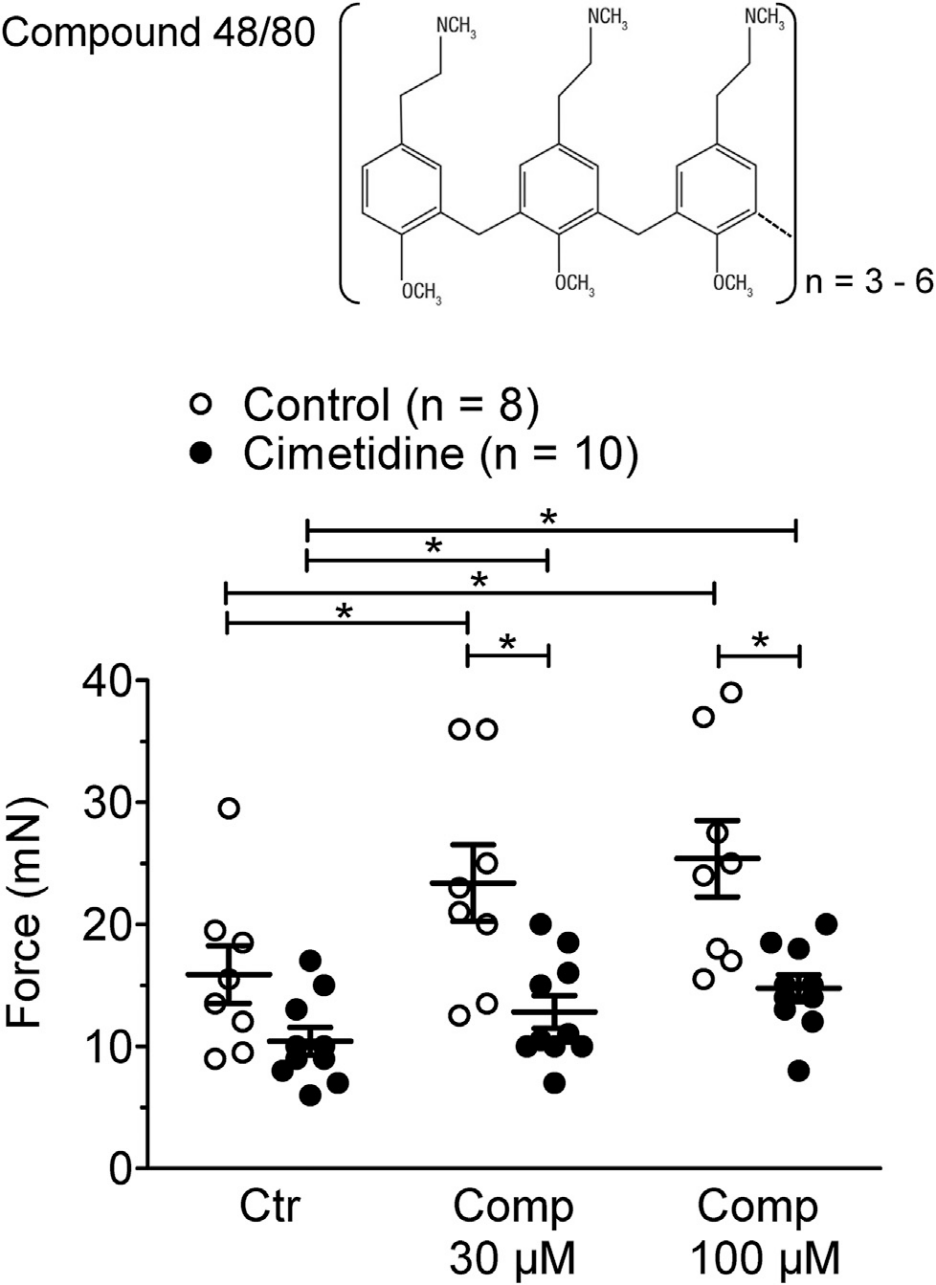
Effect of compound 48/80 alone (Comp 30 or 100 μM, open bars) or in the presence of 10 µM cimetidine (closed bars) on FOC of isolated electrically driven (1 Hz) left atrium of H_2_-receptor overexpressing H_2_-TG mice. Numbers in bars indicate the number of animals studied. *p < 0.05 vs. Ctr (no drug addition); ^**§**^ *p* < 0.05 vs. absence of compound 48/80.

In mouse atrial preparations, the histamine releasing agent compound 48/80 exerted a PIE on left atria from H_2_-TG and this effect was attenuated by cimetidine (Figure 4). Initial experiments detected a PIE of compound 48/80 also in WT atria, which was absent in the additional presence of 50 µM propranolol. Therefore, the experiments for compound 48/80 in H_2_-TG and WT all contained 50 µM propranolol. Under these conditions compound 48/80 was unable to exert a PIE in WT atria (data not shown).

### Effects of Enzyme Inhibitors: Aminoguanidine, Quinacrine and Tranylcypromine

**FIGURE 5:**
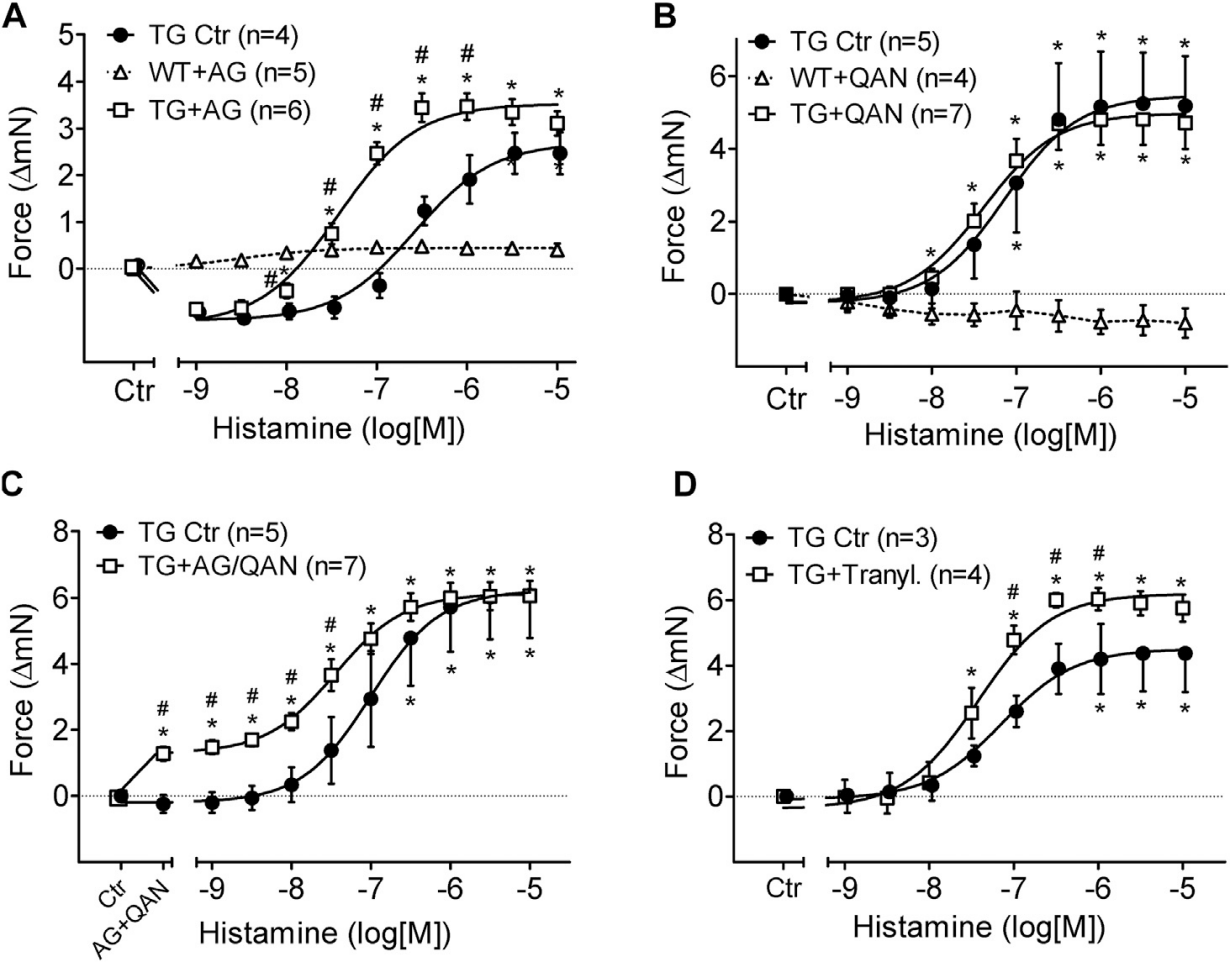
Effects of inhibitors of histamine metabolizing enzymes on FOC of isolated electrically driven (1 Hz) left atrium of H_2_-receptor overexpressing mice (TG) or wild-type controls (WT). **(A)** Effect of histamine alone (TG Ctr) or in the presence of 1 mM aminoguanidine (TG + AG). **(B)** Effect of histamine alone (TG Ctr) or in the presence of 2 µM quinacrine (TG + QAN). Here, a WT preparation (WT + QAN) is shown to demonstrate the generally missing histamine effect in WT. **(C)** Effect of histamine alone (TG Ctr) or in the combined presence of 2 µM quinacrine and 1 mM aminoguanidine (TG + AG/QAN). **(D)** Effect of histamine alone (TG Ctr) or in the combined presence of 10 µM tranylcypromine and 10 µM propranolol (TG + Tranyl.). Numbers in brackets indicate the number of animals studied. Ctr = basal contraction before drug addition. *p < 0.05 vs. Ctr; ^#^ *p* < 0.05 TG drug treated vs. TG Ctr.

Aminoguanidine (AG) alone (1 mM), an inhibitor of diamine oxidase (Figure 5A), reduced FOC in H_2_-TG and shifted the concentration response curve of histamine on FOC to the left in H_2_-TG but was ineffective in WT (Figure 5A). More specifically, aminoguanidine (1 mM) shifted the log concentration response curves for the PIE in H_2_-TG left atrium to the left from EC_50_-values (=half maximum effective drug concentrations) of 110 nM to 37 nM (n = 4–6; *p* < 0.05).

Quinacrine (QAN), an inhibitor of histamine-N-methyltransferase (HMT) that inactivates histamine (Figure 5B), did not shift the concentration response curve of exogenously administered histamine on FOC in H_2_-TG atrium and was ineffective in WT (Figure 5B). Now, one can ask what would happen if one inhibited both metabolizing pathways: diamine oxidase and histamine-N-methyltransferase: aminoguanidine combined with quinacrine (Figure 5C) increased per se FOC in H_2_-TG, suggesting an action of endogenous histamine and shifted the concentration response curve of histamine on FOC (Figure 5C) to the left in H_2_-TG but was ineffective in WT (data not shown).

Similar effects of aminoguanidine and quinacrine were noted on the beating rate of right atrial preparations from H_2_-TG. Regrettably, only a few right atria per group remained in sinus rhythm till the end of the histamine concentration response curves in the additional presence of inhibitors of metabolism (Supplementary Figure S2). Therefore, it was difficult to properly analyze the effects of the inhibitors on the beating rate, that is to say that the noted differences lack statistically significance (Supplementary Figure S2). In WT right atrial preparations, aminoguanidine, quinacrine and of course histamine were ineffective (Supplementary Figure S2).

Furthermore, the inhibitor of both monoamine oxidase A and B (MAO-A and MAO-B), tranylcypromine (10 µM), shifted the PIE of histamine in H_2_-TG atrium from EC_50_-values of 70 nM to 38 nM and increased the efficacy of histamine for the PIE in H_2_-TG atrium from a maximum response of 9.3 ± 0.8 mN to 12.4 ± 0.2 mN (n = 3–4; *p* < 0.05, Figure 5D). Experiments were performed in the presence of 50 µM of the *ß*-adrenoceptor antagonist propranolol because we noted in initial experiments a profound PIE of tranylcypromine alone (in H_2_-TG and WT). This PIE was blocked by previously applied propranolol suggesting that the PIE of tranylcypromine (in the absence of propranolol) resulted from elevated noradrenaline near the β-adrenoceptor in the sarcolemma, due to inhibition of noradrenaline uptake into cells.

### Enzyme Activity

In mouse cardiac tissue (n = 2–3), we detected similar activities of HMT in WT and H_2_-TG. Namely, in left atrium we measured HMT as 60.4 ± 0.2 pmol/min/mg protein (H_2_-TG) compared to 68.1 ± 4.7 pmol/min/mg protein (WT). In right atrium we noted HMT as 80.4 pmol/min/mg protein (H_2_-TG) compared to 114 ± 10.1 pmol/min/mg protein (WT). In ventricles we observed HMT as 83.2 ± 1.1 pmol/min/mg protein (H_2_-TG) vs. 79.1 ± 3.5 pmol/min/mg protein (WT). As concerns MAO-B, in left atrium we detected 1147 pmol/min/mg (H_2_-TG) and 1759 pmol/min/mg (WT) and in right atrium 879 pmol/min/mg (H_2_-TG) vs. 1056 ± 152 pmol/min/mg (WT). In ventricles, MAO-B amounted to 903 ± 50.7 pmol/min/mg (H_2_-TG) and 869 ± 27.6 pmol/min/mg (WT). Interestingly, DAO activity was very low amounting in ventricles and amounted to 1.08 pmol/min/mg (H_2_-TG) and 1.45 pmol/min/mg (WT). In atrial samples, DAO activity was below our level of detection due to the small size of the murine atrium. In human atrial samples recovered during surgery, we could measure MAO-A and MAO-B activity of 666 ± 166 and 891 ± 35.7 pmol/min/mg protein (n = 3), respectively, and histamine concentration in these samples amounted to 845 ± 60.5 ng/g (n = 3).

### Western Blotting and Histology

**FIGURE 6:**
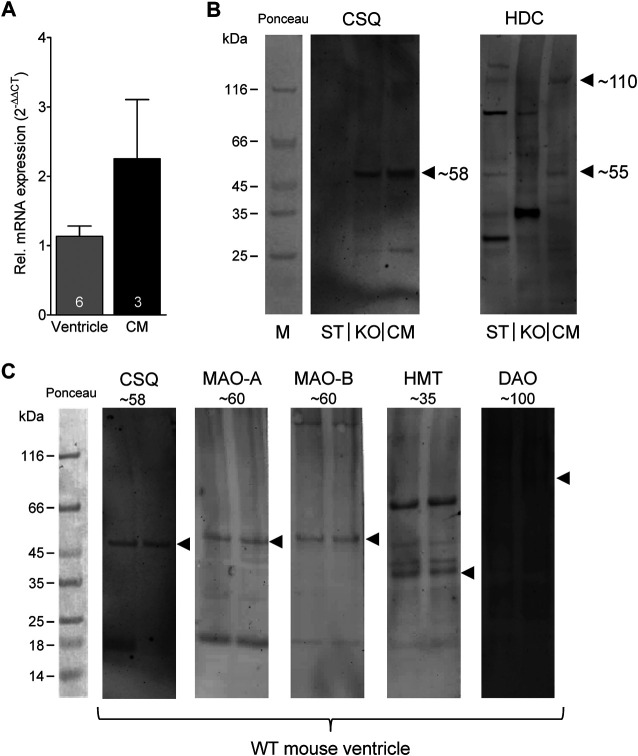
Detection of histamine metabolizing enzymes. **(A)** By real time reverse transcription PCR, expression of HDC mRNA could be detected in mouse ventricular samples as well as in isolated cardiac myocytes (CM). Numbers in bars indicate number of animals studied. **(B)** Representative Western blots for HDC demonstrate its presence in isolated mouse cardiac myocytes (CM) in its active 53 kDa form (note the homodimer at about 110 kDa). A mouse stomach sample (ST) was used as positive control and a cardiac sample of a HDC knockout mouse (KO) as negative control. Cardiac calsequestrin (CSQ) was used as loading control especially for cardiac samples (note the missing band in ST). **(C)** Representative Western blots for CSQ (loading control), MAO-A, MAO-B, HMT and DAO (extremely low amounts) of mouse tissue homogenates. The corresponding molecular weight of each enzyme is indicated. For better orientation, the ponceau stained molecular weight marker is shown.

**FIGURE 7:**
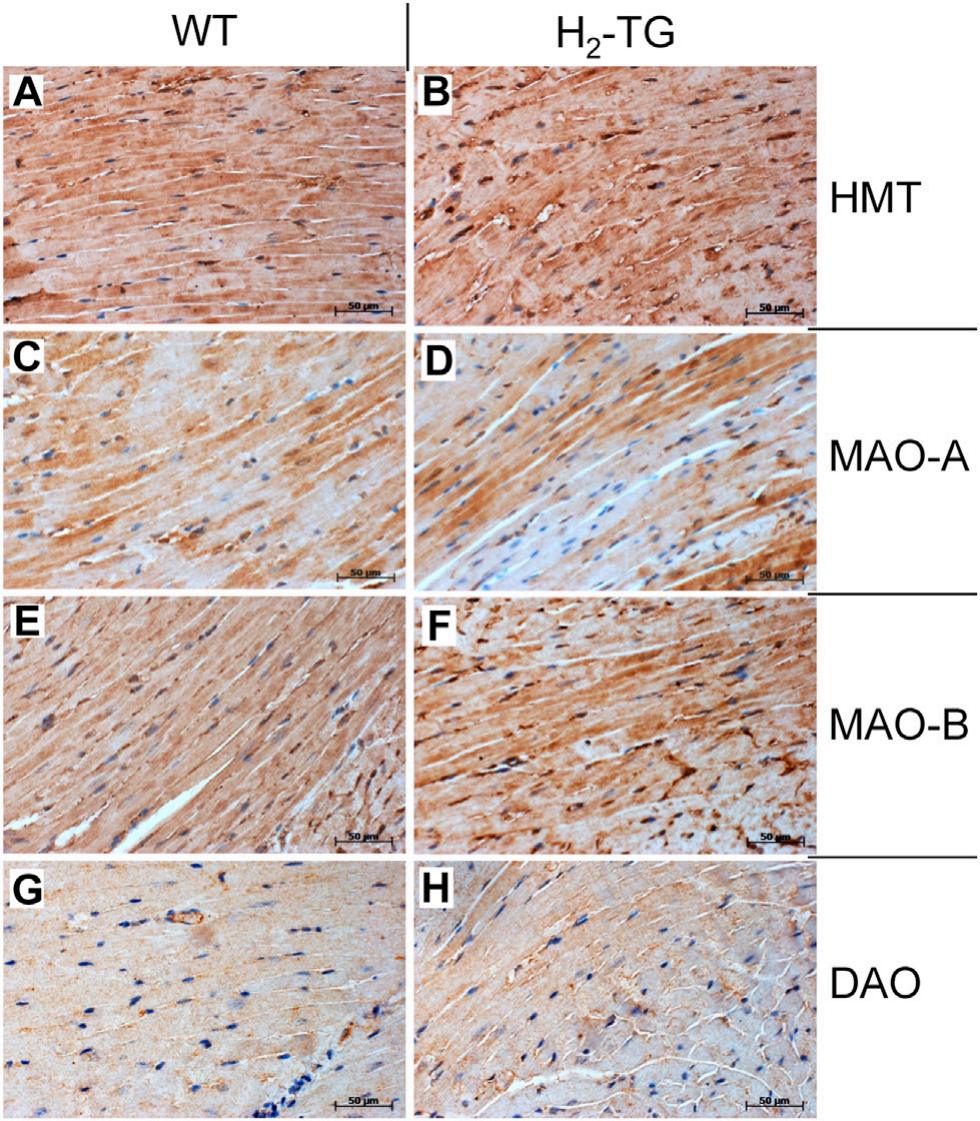
Immunohistochemical detection of histamine-metabolizing enzymes in the mouse myocardium. The localization of histamine-N-methyltransferase (HMT, **A,B**), monoamine oxidase A (MAO-A, **C,D**) and B (MAO-B, **E,F**) as well as diamine oxidase (DAO, **G,H**) in the myocardium of H_2_-TG mice and wild-type mice (WT) is shown. Immunocomplexes were detected by DAB-HRP staining (brown staining) and nuclei were visualized by counterstaining with haematoxylin. Horizontal bars indicate 50 µm.

**FIGURE 8:**
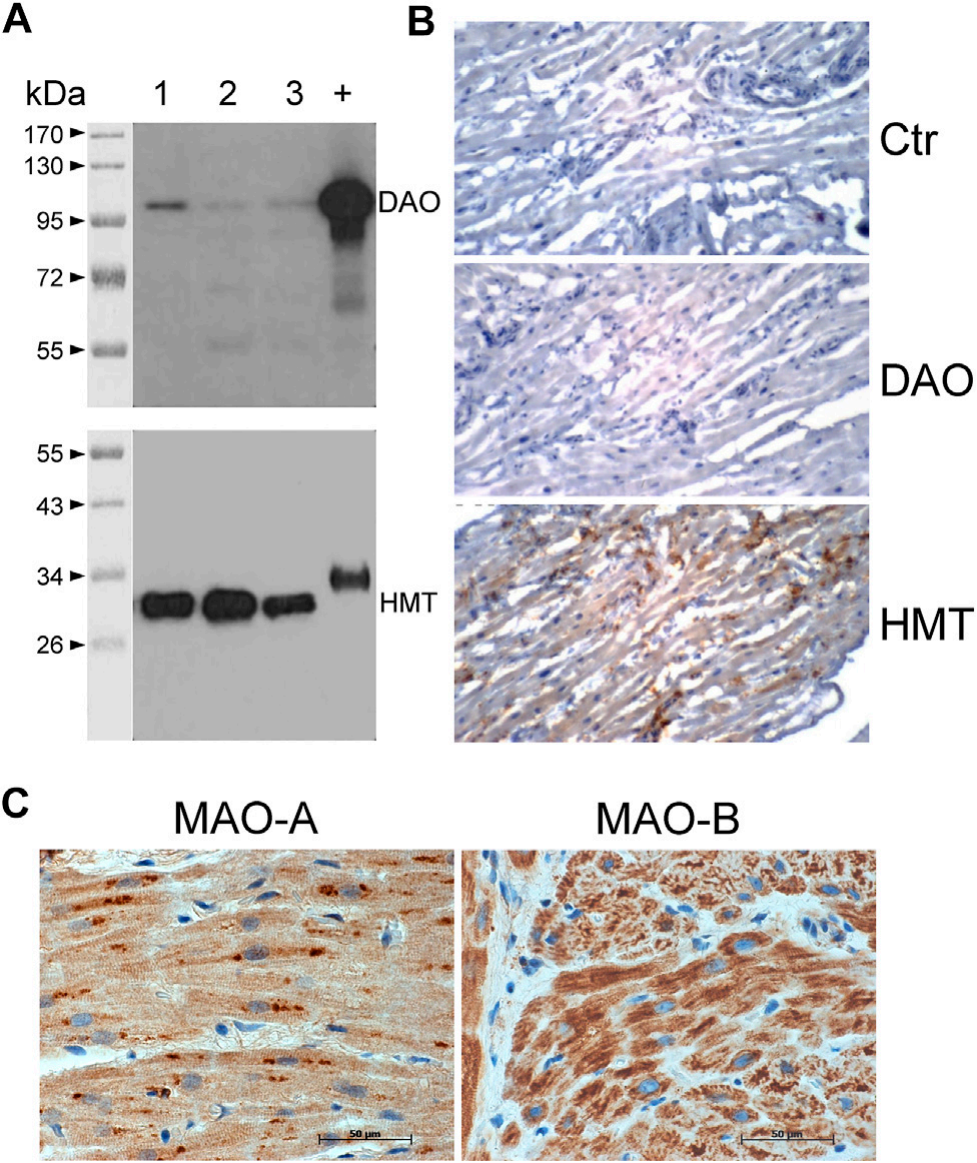
Detection of histamine-metabolizing enzymes in human atria by immunohistochemical staining and immunoblotting. **(A)** Diamine oxidase (DAO, 105 kDa) and histamine-N-methyltransferase (HMT, 33 kDa) were detected on blots of 10 µg total protein from human atrial homogenates from three patients (lanes 1–3) separated on 10% (DAO) or 12.5% (HMT) SDS polyacrylamide gels using specific monoclonal antibodies. +, positive control of 400 ng human kidney homogenate; kDa, molecular weight markers with sizes of proteins in kDa. **(B)** Immunohistochemical stainings of DAO and HMT on thin sections of human atrial specimens are presented. Sections were incubated without specific antibody (Ctr) or with specific monoclonal antibodies for DAO and HMT. **(C)** Immunohistochemical localization of MAO-A and MAO-B in the human atrial myocardium. **(B,C)** Immunocomplexes were detected by DAB-HRP staining (brown staining) followed by counterstaining of nuclei with haematoxylin. Horizontal bars indicate 50 µm.

Given the effects of the various inhibitors described above, we asked if the different enzymes presumably inhibited by these compounds are present in mouse and human heart tissue. We could detect the mRNA of HDC in mouse ventricular samples and in isolated mouse cardiac myocytes (Figure 6A) and the HDC protein in its active 53 kDa form in isolated mouse cardiac myocytes (Figure 6B). Here, a mouse stomach sample was used as positive control and a cardiac sample of a HDC knockout mouse as negative control (Figure 6B). By comparison with the knockout sample, HDC was detected as monomer and dimer as indicated. The detection of cardiac calsequestrin (CSQ) was used as loading control especially for cardiac samples (Figure 6B). Moreover, we could detect MAO-A, MAO-B, DAO (extremely low amounts) and HMT on Western blots of mouse tissue homogenates (exemplary blots are shown in Figure 6C). In addition, we were able to detect and localize the HMT, MAO-A, MAO-B and, to considerably lesser extent, DAO in mouse cardiac tissue, specifically in cardiomyocytes (Figure 7), using immunohistology. Likewise, we could detect HMT and trace amounts of DAO in human atrial tissue by Western blotting (Figure 8A), and HMT, MAO-A and MAO-B but not DAO in human atrium by immunohistological staining (Figures 8B,C).

**FIGURE 9:**
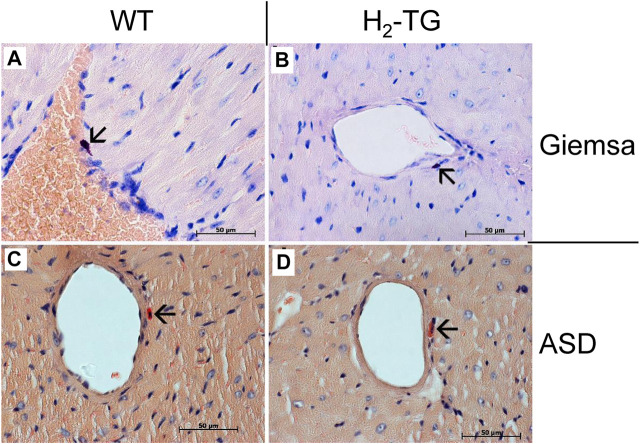
Analyses of mast cells in the mouse myocardium. Localization of mast cells was performed by Giemsa stain **(A,B)** of the myocardium of WT mice **(A)** and H_2_-TG mice **(B)** as well as by ASD-Chloracetate-Esterase activity **(C,D)**, characteristic for mast cells, in the myocardium of WT mice **(C)** and H_2_-TG mice **(D)**. These representative pictures demonstrate the rare occurrences of mast cells in the mouse myocardium. Mast cells are marked with arrows. Nuclei are counterstained with haematoxylin. Horizontal bars indicate 50 µm.

**FIGURE 10:**
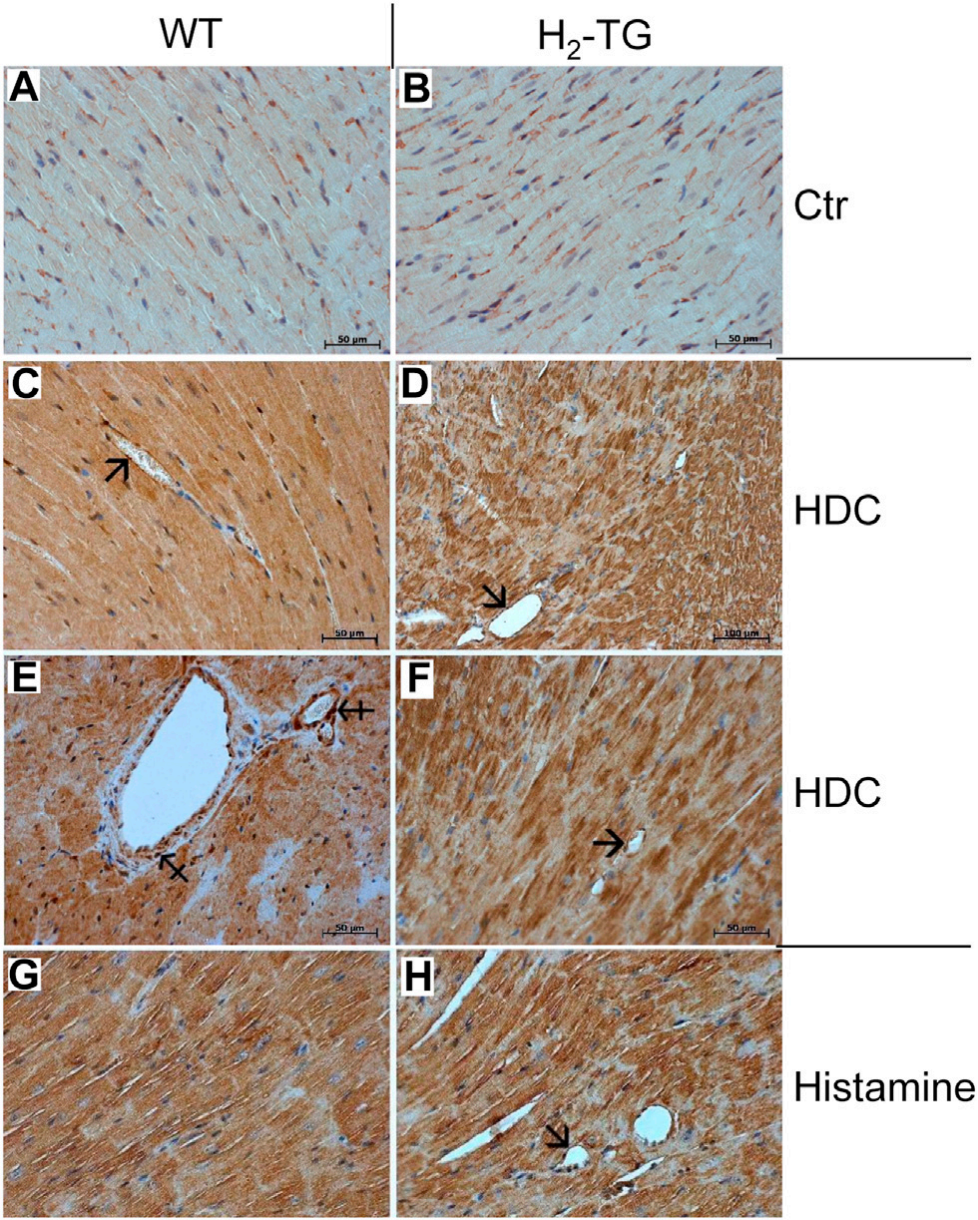
Immunohistochemical localization of histidine decarboxylase (HDC) and histamine in the mouse myocardium. **(A,B)** Negative controls (Ctr) of WT and H_2_-TG samples. HDC could be localized in the myocardium of WT mice **(C,E)** and in the myocardium of H_2_-TG mice **(D,F)**. Positive immunostaining was observed in the cardiomyocytes as well as in the endothelium (arrows) and in the vascular smooth muscle cells (tailed arrows) of blood vessels **(G,H)** Immunohistochemical localization of histamine in the myocardium of WT **(G)** and H_2_-TG **(H)** mice. Along with cardiomyocytes, positive immunostaining can also be seen in the endothelium (arrow). Immunocomplexes were detected by DAB-HRP staining (brown staining) followed by counterstaining of nuclei with haematoxylin. Horizontal bars indicate 50 µm.

Classically, the mast cells are a possible source of histamine. Identification of mast cells in the myocardium by Giemsa stain (Figures 9A,B) as well as by an enzymohistochemical reaction for visualization of specific ASD-chloracetate-esterase activity (Figures 9C,D), permitted to reveal only extremely rare mast cells exclusively in the close vicinity of blood vessels both in WT- (Figures 9A,C) and in H_2_-TG-samples (Figures 9B,D). Control stainings for mast cells (positive controls) were performed on samples of a human mastocytosis case and a gastrointestinal stromal tumor case (data not shown). Interestingly, in all WT- and H_2_-TG-samples from mice, positive immunostaining was observed for histidine decarboxylase (Figure 10) in atria. Histidine decarboxylase was immunostained in cardiomyocytes as well as in intima and media of blood vessels (Figures 10C–F). The histamine produced can be made visible histologically in the mouse heart. In all WT- and H_2_-TG-samples, positive immunostaining for histamine was observed. Of note, histamine was immunostained also in cardiomyocytes (Figures 10G,H).

Moreover, by mass spectrometry we could measure histamine in mouse ventricular myocytes at a concentration range of 0.0128–0.0190 pmol/mg protein. In mouse hearts, freshly taken out from mice (where blood was not washed out) the histamine content amounted to 0.177 pmol/mg heart tissue and was much lower after Langendorff perfusion with Tyrode’s solution namely 0.038 pmol/mg heart tissue. In blood of mice, we typically measure histamine concentrations about 39 pmol/ml and in serum about 16.5 pmol/ml.

## Discussion

Histidine is taken up in the gut from food by a transporter. At least in the central nervous system it enters the nerve cells, is converted to histamine by HDC, and this histamine is stored and released by the nerve cells where it acts on histamine receptors in an autocrine or paracrine fashion (Review: Haas et al., 2008; Panula et al., 2015). A similar mechanism was found in a linage of hematopoietic stem cells, which produce histamine by HDC and where histamine acts in an auto/paracrine fashion via H_2_ receptors to protect them from myelotoxic injury (Chen et al., 2017a). We assume that also in cardiomyocytes this transporter passes histidine trough the sarcolemma and, in the cytosol, it can be a substrate for HDC. Possibly, this transporter is differentially expressed in human and mouse cardiac tissues or HDC expression itself is different. Moreover, one could argue that only little histidine (a polar molecule) might pass via diffusion in the cell and the transport of histidine might be too weak to transport substantial histidine amounts into the cell. Further, a loss of the histamine formed in the cell may occur because it may be rapidly degraded to inotropically inactive metabolites or the outward transport systems of histamine (which exist) might be insufficient to transport sufficiently high concentrations of histamine out of the cell where is can bind to the ligand binding site on the outer surface of the sarcolemma. That all would be an explanation for the small effect of histidine compared to exogenously applied histamine and for the smaller effect of histidine in mouse left atrium compared to human right atrium. But this is currently speculative. Much larger concentrations of exogenously applied histidine are required to achieve a PIE than for histamine. That is plausible as first HDC has to form histamine from histidine and the formed histamine has to exit the sarcolemma, as the ligand binding site of histamine receptors is located on the outer surface of the sarcolemma. These findings are reminiscent of our previous work where we noted for another monoamine, 5-HT, that it can be formed by decarboxylation from its direct precursor (5-hydroxy-tryptophane) in the heart and can exert a PIE in the isolated human atrium (Gergs et al., 2017b).

HDC is certainly the main histamine producing enzyme at least in mice, because HDC-KO mice have very low levels of histamine in tissue and plasma (Ohtsu et al., 2001; review:; Jutel et al., 2009). Most mammalian blood cells and some neuronal cells contain HDC and thus can form histamine (Jutel et al., 2009). HDC is classically found in mast cells, basophils and enterochromaffin-like cells where it is constitutively expressed (Jutel et al., 2009; Fogel, 2015). During inflammation, initiated by LPS, HDC expression seems to be induced in the lung and stomach but in this experimental model, the HDC promoter was hardly active in the heart. These expression patterns were identified by green fluorescence protein expression under control of the HDC promoter in transgenic mice (Takai et al., 2019). In the human heart, HDC was imunohistologically detected in half of the ganglions of the right atrium (Singh et al., 1999) and in isolated rat cardiomyocytes, HDC was detected by Western blotting (Costiniti et al., 2018). In the present paper, we present the detection of HDC in mouse cardiomyocytes by immunohistology, Western blotting und real time RT-PCR. Cardiac HDC expression in mice may be strain-dependent but, furthermore, an important role for histamine in the heart was demonstrated by a mouse model of myocardial infarction using HDC-KO mice. In these mice, histamine deficiency worsened cardiac dysfunction (Deng et al., 2015; Chen et al., 2017b). But by use of HDC-EGFP transgenic mice, it was shown that CD11b and Gr-1 positive immature myeloid cells seem to be the predominant HDC-expressing sites in acute myocardial infarction (Deng et al., 2015). This source of HDC, more precisely, HDC-expressing macrophages located in the mouse heart, may also apply in our model, but we investigated the basal expression pattern of HDC. Nevertheless, this is a limitation of our study. Therefore, it would be interesting to investigate by use of HDC-EGFP transgenic mice if EGFP expression can be found in cardiomyocytes under basal conditions. Moreover, it would be interesting to study whether any HDC activity is present in isolated cardiomyocytes. Unfortunately, these experiments are beyond the scope of our current study.

Inactive mutants of HDC have been reported and linked to neurological diseases like Tourette`s syndrome (Jones and Kearns, 2011). These patients should have less histamine in the heart but apparently this has not been reported so far. HDC was detected by Western blotting in rabbit atrium (Matsuda et al., 2002). HDC is also expressed by some bacteria in the gut (Chen et al., 2019) but also some fermented food (cheese, red wine) and fish may contain substantial levels of histamine and can lead to adverse reactions (Maintz et al., 2006). Once histamine activates the H_2_-receptor, a cascade leading to phospholamban (PLB) phosphorylation is initiated (Figure 1), which we could detect in human atrial preparations and which can explain at least in part the inotropic as well as the lusitropic effects of histamine in the human heart in agreement with our findings in H_2_-TG atrium (Gergs et al., 2019a). Moreover, histamine can regulate the beating rate via H_2_-receptors of the vertebrate heart (reptile) but the origin of the histamine was not evaluated (Skovgaard et al., 2009).

We present tentative evidence that histamine can be released from the H_2_-TG atrium, as compound 48/80 exerts a cimetidine sensitive effect (Figure 4). It is well known that compound 48/80 can extrude histamine from the heart (guinea pig heart: Gomez and Antonio, 1981; human heart: Patella et al., 1995). Unfortunately, it is currently unclear from which cell type histamine is released in H_2_-TG atrium by compound 48/80. On the one hand, it is assumed that muscle cells, likewise including cardiac muscle cells, cannot store histamine (Fogel, 2015) but on the other hand, mast cells, a main source of histamine, are extremely rare in the mouse atrium as we noted here. So far, this question remains unanswered.

Histamine N-methyltransferase (HMT) converts and inactivates histamine by forming 1-methyl-histamine (Ganellin, 1982). HMT was found within liver cells and is thought to degrade intracellular histamine (Brown et al., 1959; Yoshikawa et al., 2013). At least in the guinea pig heart and pig hearts, HMT was detected earlier (Tahara et al., 2000; Klocker et al., 2005). HMT can be inhibited by diphenhydramine and other histamine receptor antagonists which might pose problems in data interpretation (Adachi et al., 2011). In the rat brain, HMT inhibitors could increase the levels of histamine *in vivo* (Duch et al., 1978), but which is difficult to compare with our present findings. It is suggested that HMT is localized intracellular and histamine has to be transported into the cytosol before it can be methylated by HMT (Ogasawara et al., 2006). Therefore, one could speculate that the role of HMT for degradation of exogenously administered histamine in the mouse heart *in vitro*, like in our experiments, depends also on the activity of the cardiac monoamine transporters. Moreover, the uptake of exogenously applied histamine into the cells might be slower in the heart than in the brain and species differences should be considered. All this might explain the missing effect of HMT inhibition in isolated atria. This needs to be clarified. There are mutations of HMT in patients with reduced enzymatic activity and reduced stability (Heidari et al., 2015). These patients should have higher levels of histamine in the heart leading to arrhythmias and hypotension (higher histamine levels in vessels) judged from our present work. But this hypothesis requires testing. In all regions of the mouse heart and (as a novel finding) in human atrium, we noted HMT using enzymatic assays, Western blotting or immunohistochemistry. No differences in activities between H_2_-TG and WT were noticeable.

Typically, extracellular histamine will be degraded by DAO. At least in the intestinal tract, DAO is released by enterocytes and can destroy histamine present in food, possibly a protective mechanism (Mainz et al., 2006). DAO is thought not to be expressed in the central nervous system but in the periphery (Yoshikawa et al., 2013). Nevertheless, it was shown that DAO can be present under physiological conditions in the plasma of mouse and man (Schwelberger, 2010) and DAO was detected earlier in the mouse heart (Bono et al., 1999) but not e.g. in pig hearts (Klocker et al., 2005) indicating relevant species differences. Exogenous histamine increased activity of DAO in the plasma, supposedly by a negative feedback loop (Wollin et al., 1998). One might argue that we detected only minute amounts of DAO in the heart that might be insignificant for histamine inactivation. Therefore, the effect of DAO might be indirect and in the future it might be possible to resolve this issue by use of DAO knock-out mice. Aminoguanidine is a classical inhibitor of the activity of DAO (Sattler et al., 1985). However, clinically utilized drugs (with totally different chemical structures) like the antihypertensive drug dihydralazine, the anti-malarial drug chloroquine, the antibiotic cycloserine and the muscle relaxant tubocurine (Sattler et al., 1985), but also cimetidine, diphenhydramine and ethanol (Wantke et al., 1998) can inhibit the activity of DAO. Thus, in some patients these drugs might inadvertently increase histamine levels in the human heart and might thus likewise lead to cardiac arrhythmias (as histamine, like all cAMP increasing drugs, is known to induce arrhythmias in some patients: Wolff and Levi, 1986; atrial fibrillation: Layritz et al., 2014). At least in pigs, drastic hypotension (as a result of histamine induced vasodilation) was noted when animals were given 60 mg histamine per os in the additional presence of aminoguanidine (Sattler et al., 1988). Mutations of DAO have been reported which are implied in gastrointestinal diseases (Maintz et al., 2006; Jones and Kearns, 2011). It would be interesting to study cardiac function after histamine infusion in such patients to find out whether these mutations might induce arrhythmias by increasing histamine levels in the heart. Moreover, in clinical studies an increase in cardiac levels of histamine correlated with the presence of heart failure (e.g. Patella et al., 1998). This might be a compensatory effect like the increase in noradrenaline in heart failure patients, in order to sustain cardiac force of contraction. High levels of histamine in an animal model increased the incidence of myocardial infarction (Dai, 1976). Of note, the activity of DAO was very small in mouse cardiac tissue and human atrial tissue whereas the effect of aminoguanidine on the PIE of histamine is quite pronounced (Figure 5). This apparent discrepancy might indicate that the localization of DAO on the cell surface (in this case the sarcolemma) might be strategically important to diminish the level of histamine on the crucial histamine receptor outer surface. But this remains speculative.

The degradation product of histamine called 1-methylhistamine is oxidized in mitochondria by MAO-B (Waldmeier et al., 1977). In human hearts, about similar levels of MAO-A and MAO-B were immunnologically detected (Duicu et al., 2015). Rat hearts contain MAO-A and -B in a ratio of 100:1 (Saura et al., 1992). Functionally, the rat heart mainly expresses MAO-A whereas the mouse heart mainly expresses MAO-B (Dorris, 1982). That indicates that we probably have inhibited mainly MAO-B in our mouse model with tranylcypromine and indicates that the contractile alterations in the presence of tranylcypromine might be due to inhibition of MAO-B in H_2_-TG atrium. Using Western blotting, enzyme activity measurement and immunohistology we could detect high expression and activity of MAO-B in all regions of the mouse heart investigated as well as in human atrial samples. However, one might argue that the importance of MAO-B remains unclear as inhibition of the activity of MAO-B would only increase 1-methylhistamine levels but not necessarily histamine levels in the heart (Figure 2). Thus, we cannot rule out indirect effects of tranylcypromine.

## Conclusion

In conclusion, the data in this study indicate that exogenously applied histidine can be converted to inotropically active histamine, which is then subject to degradation in the mammalian heart. But further studies are necessary to clearly localize the source of histamine and the pathway of degradation in the heart.

## Data Availability

The raw data supporting the conclusion of this article will be made available by the authors, without undue reservation.

## References

[B1] Ackermann D. (1910). Über den bakteriellen Abbau des Histidins. Hoppe-Seyler´s Z. für physiologische Chem. 65, 504–510. 10.1515/bchm2.1910.65.5-6.504

[B2] Adachi N. Liu K. Ninomiya K. Matsuoka E. Motoki A. Irisawa Y. (2011). Reduction of the Infarct Size by Simultaneous Administration of L-Histidine and Diphenhydramine in Ischaemic Rat Brains. Resuscitation 82 (2), 219–221. 10.1016/j.resuscitation.2010.10.024 21131122

[B3] Anton A. H. Sayre D. F. (1969). A Modified Fluorometric Procedure for Tissue Histamine and its Distribution in Various Animals. J. Pharmacol. Exp. Ther. 166 (2), 285–290. 5776986

[B4] Baumann G. Felix S. B. Rie G. Loher U. Ludwig L. Blömer H. (1982). Effective Stimulation of Cardiac Contractility and Myocardial Metabolism by Impromidine and Dimaprit-Two New H2-Agonistic Compounds-In the Surviving, Catecholamine-Insensitive Myocardium after Coronary Occlusion. J. Cardiovasc. Pharmacol. 4 (4), 542–543. 10.1097/00005344-198207000-00004 6181327

[B5] Baumann G. Mercader D. Busch U. Felix S. B. Loher U. Ludwig L. (1983). Myocardium from Patients with Heart Failure Due To Mitral and Aortic Valve Disease. J. Cardiovasc. Pharmacol. 5 (4), 618–625. 10.1097/00005344-198307000-00017 6193360

[B6] Baumann G. Permanetter B. Wirtzfeld A. (1984). Possible Value of H2-Receptor Agonists for Treatment of Catecholamine-Insensitive Congestive Heart Failure. Pharmacol. Ther. 24 (2), 165–177. 10.1016/0163-7258(84)90033-0 6087383

[B7] Boknik P. Drzewiecki K. Eskandar J. Gergs U. Hofmann B. Treede H. (2019). Evidence for Arrhythmogenic Effects of A2A-Adenosine Receptors. Front. Pharmacol. 10, 1051. 10.3389/fphar.2019.01051 31619997 PMC6759833

[B8] Bono P. Jalkanen S. Salmi M. (1999). Mouse Vascular Adhesion Protein 1 Is a Sialoglycoprotein with Enzymatic Activity and Is Induced in Diabetic Insulitis. Am. J. Pathol. 155 (5), 1613–1624. 10.1016/s0002-9440(10)65477-6 10550318 PMC1866981

[B9] Bradford M. M. (1976). A Rapid and Sensitive Method for the Quantitation of Microgram Quantities of Protein Utilizing the Principle of Protein-Dye Binding. Anal. Biochem. 72, 248–254. 10.1016/0003-2697(76)90527-3 942051

[B11] Brown D. D. Tomchick R. Axelrod J. (1959). The Distribution and Properties of a Histamine-Methylating Enzyme. J. Biol. Chem. 234, 2948–2950. 10.1016/s0021-9258(18)69701-7 13804910

[B12] Buchwalow I. B. Boecker W. (2010). Immunohistochemistry: Basics and Methods. Heidelberg, London, United Kingdom: Springer. 10.1007/978-3-642-04609-4

[B13] Buchwalow I. Samoilova V. Boecker W. Tiemann M. (2011). Non-specific Binding of Antibodies in Immunohistochemistry: Fallacies and Facts. Sci. Rep. 1, 28. 10.1038/srep00028 22355547 PMC3216515

[B14] Buchwalow I. Schnekenburger J. Tiemann K. Samoilova V. Bankfalvi A. Poremba C. (2013). L-arginine-NO-cGMP Signalling Pathway in Pancreatitis. Sci. Rep. 3, 1899. 10.1038/srep01899 23712581 PMC3664897

[B15] Chen H. Nwe P.-K. Yang Y. Rosen C. E. Bielecka A. A. Kuchroo M. (2019). A Forward Chemical Genetic Screen Reveals Gut Microbiota Metabolites That Modulate Host Physiology. Cell 177 (5), 1217–1231. 10.1016/j.cell.2019.03.036 31006530 PMC6536006

[B16] Chen J. Hong T. Ding S. Deng L. Abudupataer M. Zhang W. (2017a). Aggravated Myocardial Infarction-Induced Cardiac Remodeling and Heart Failure in Histamine-Deficient Mice. Sci. Rep. 7, 44007. 10.1038/srep44007 28272448 PMC5341031

[B17] Chen X. Deng H. Churchill M. J. Luchsinger L. L. Du X. Chu T. H. (2017b). Bone Marrow Myeloid Cells Regulate Myeloid-Biased Hematopoietic Stem Cells via a Histamine-Dependent Feedback Loop. Cell Stem Cell 21 (6), 747–760. 10.1016/j.stem.2017.11.003 29198940 PMC5975960

[B18] Costiniti V. Spera I. Menabò R. Palmieri E. M. Menga A. Scarcia P. (2018). Monoamine Oxidase-dependent Histamine Catabolism Accounts for Post-ischemic Cardiac Redox Imbalance and Injury. Biochim. Biophys. Acta (Bba) - Mol. Basis Dis. 1864 (9 Pt B), 3050–3059. 10.1016/j.bbadis.2018.06.018 29953926

[B19] Dai S. (1976). A Study of the Actions of Histamine on the Isolated Rat Heart. Clin. Exp. Pharmacol. Physiol. 3 (4), 359–367. 10.1111/j.1440-1681.1976.tb00612.x 975624

[B20] Dale H. H. Laidlaw P. P. (1910). The Physiological Action of β-iminazolylethylamine. J. Physiol. 41 (5), 318–344. 10.1113/jphysiol.1910.sp001406 16993030 PMC1512903

[B21] Deng L. Hong T. Lin J. Ding S. Huang Z. Chen J. (2015). Histamine Deficiency Exacerbates Myocardial Injury in Acute Myocardial Infarction through Impaired Macrophage Infiltration and Increased Cardiomyocyte Apoptosis. Sci. Rep. 5, 13131. 10.1038/srep13131 26278136 PMC4642534

[B22] Dorris R. L. (1982). A Simple Method for Screening Monoamine Oxidase (MAO) Inhibitory Drugs for Type Preference. J. Pharmacol. Methods 7 (2), 133–137. 10.1016/0160-5402(82)90025-0 7098495

[B23] Duch D. S. Bowers S. W. Nichol C. A. (1978). Elevation of Brain Histamine Levels by Diaminopyrimidine Inhibitors of Histamine N-Methyl Transferase. Biochem. Pharmacol. 27 (10), 1507–1509. 10.1016/0006-2952(78)90109-0 697892

[B24] Duicu O. M. Lighezan R. Sturza A. Ceausu R. A. Borza C. Vaduva A. (2015). Monoamine Oxidases as Potential Contributors to Oxidative Stress in Diabetes: Time for a Study in Patients Undergoing Heart Surgery. Biomed. Res. Int. 2015, 515437. 10.1155/2015/515437 26101773 PMC4458524

[B25] Eckel L. Gristwood R. W. Nawrath H. Owen D. A. Satter P. (1982). Inotropic and Electrophysiological Effects of Histamine on Human Ventricular Heart Muscle. J. Physiol. 330, 111–123. 10.1113/jphysiol.1982.sp014332 6294285 PMC1225289

[B26] Fogel W. A. (2015). “Histamine”, In Encyclopedia of Inflammatory Diseases. Editors Parnham M., (Basel: Springer), 1–12. 10.1007/978-3-0348-0620-6_42-1

[B27] Fogel W. A. Ulatowska M. Adach K. Osińska Z. (1985). A Sum of ^14^C-Putrescine Metabolites as a Measure of DAO Activity. Column Chromatography Assay. Agents Actions 16 (3-4), 99–101. 10.1007/bf01983111 3925737

[B28] Fowler C. J. Tipton K. F. (1982). Deamination of 5-hydroxytryptamine by Both Forms of Monoamine Oxidase in the Rat Brain. J. Neurochem. 38 (3), 733–736. 10.1111/j.1471-4159.1982.tb08692.x 7057191

[B29] Ganellin C. R. (1982). “Chemistry and Structure-Activity Relationships of Drugs Acting at Histamine Receptors”, In Pharmacology of Histamine Receptors. Editors Hanellin C. R. Parsons M. E. (Bristol: Wright), 10–102. 10.1016/b978-0-7236-0589-8.50008-9

[B30] Gergs U. Baumann M. Böckler A. Buchwalow I. B. Ebelt H. Fabritz L. (2010). Cardiac Overexpression of the Human 5-HT4 Receptor in Mice. Am. J. Physiology-Heart Circulatory Physiol. 299 (3), H788–H798. 10.1152/ajpheart.00691.2009 20639221

[B31] Gergs U. Böckler A. Ebelt H. Hauptmann S. Keller N. Otto V. (2013). Human 5-HT4 Receptor Stimulation in Atria of Transgenic Mice. Naunyn-schmiedeberg's Arch. Pharmacol. 386 (5), 357–367. 10.1007/s00210-013-0831-x 23307014

[B32] Gergs U. Grobe J. M. Neumann J. (2016). Diamine Oxidase and Monoamine Oxidase Can Degrade Histamine in the Mammalian Heart to an Inotropically Relevant Extent. Inflamm. Res. 65 (Suppl. 1), S52. 10.1007/s00011-016-0958-6

[B33] Gergs U. Fritsche J. Fabian S. Christ J. Neumann J. (2017a). Desensitization of the Human 5-HT4 Receptor in Isolated Atria of Transgenic Mice. Naunyn-schmiedeberg's Arch. Pharmacol. 390 (10), 987–996. 10.1007/s00210-017-1403-2 28689254

[B34] Gergs U. Jung F. Buchwalow I. B. Hofmann B. Simm A. Treede H. (2017b). Pharmacological and Physiological Assessment of Serotonin Formation and Degradation in Isolated Preparations from Mouse and Human Hearts. Am. J. Physiology-Heart Circulatory Physiol. 313 (6), H1087–H1097. 10.1152/ajpheart.00350.2017 28916638

[B35] Gergs U. Bernhardt G. Buchwalow I. B. Edler H. Fröba J. Keller M. (2019a). Initial Characterization of Transgenic Mice Overexpressing Human Histamine H2 Receptors. J. Pharmacol. Exp. Ther. 369, 129–141. 10.1124/jpet.118.255711 30728249

[B36] Gergs U. Trapp T. Bushnaq H. Simm A. Silber R.-E. Neumann J. (2019b). Age-Dependent Protein Expression of Serine/Threonine Phosphatases and Their Inhibitors in the Human Cardiac Atrium. Adv. Med. 2019, 1–9. 10.1155/2019/2675972 PMC6334353 30719459

[B37] Gergs U. Jahn T. Werner F. Köhler C. Köpp F. Großmann C. (2019c). Overexpression of Protein Phosphatase 5 in the Mouse Heart: Reduced Contractility but Increased Stress Tolerance - Two Sides of the Same Coin? PloS one 14 (8), e0221289. 10.1371/journal.pone.0221289 31425567 PMC6699691

[B38] Ginsburg R. Bristow M. R. Stinson E. B. Harrison D. C. (1980). Histamine Receptors in the Human Heart. Life Sci. 26 (26), 2245–2249. 10.1016/0024-3205(80)90209-x 6105606

[B39] Gomes J. C. Antonio A. (1981). Effects of Compound 48/80 on the guinea-pig Isolated Heart: a Comparison with the In Vitro Cardiac Anaphylaxis. Pharmacol. Res. Commun. 13 (9), 873–890. 10.1016/s0031-6989(81)80047-1 7335764

[B40] Grobe J. Gergs U. Neumann J. (2016). Functional Studies on Histamine Metabolism in the Mammalian Heart. Naunyn Schmiedebergs Arch. Pharmacol. 389 (Suppl. 1), S38. 10.1007/s00210-016-1213-y

[B41] Haas H. L. Sergeeva O. A. Selbach O. (2008). Histamine in the Nervous System. Physiol. Rev. 88 (3), 1183–1241. 10.1152/physrev.00043.2007 18626069

[B42] Hattori Y. Gando S. Endou M. Kanno M. (1991). Characterization of Histamine Receptors Modulating Inotropic and Biochemical Activities in Rabbit Left Atria. Eur. J. Pharmacol. 196 (1), 29–36. 10.1016/0014-2999(91)90405-f 1651868

[B43] Hattori Y. Nakaya H. Endou M. Kanno M. (1990). Inotropic, Electrophysiological and Biochemical Responses to Histamine in Rabbit Papillary Muscles: Evidence for Coexistence of H1- and H2-Receptors. J. Pharmacol. Exp. Ther. 253 (1), 250–256. 2158545

[B44] Hattori Y. Sakuma I. Kanno M. (1988). Differential Effects of Histamine Mediated by Histamine H1- and H2-Receptors on Contractility, Spontaneous Rate and Cyclic Nucleotides in the Rabbit Heart. Eur. J. Pharmacol. 153 (2-3), 221–229. 10.1016/0014-2999(88)90609-7 2846318

[B45] Heidari A. Tongsook C. Najafipour R. Musante L. Vasli N. Garshasbi M. (2015). Mutations in the histamineN-Methyltransferase gene,HNMT, Are Associated with Nonsyndromic Autosomal Recessive Intellectual Disability. Hum. Mol. Genet. 24 (20), 5697–5710. 10.1093/hmg/ddv286 26206890 PMC4581600

[B46] Jäger F. (1913a). Ein neuer, für die Praxis brauchbarer Sekaleersatz (Tenosin). Münchner Medizinische Wochenschrift 31, 1714–1715.

[B47] Jäger F. (1913b). Versuche zur Verwendung des β-Imidazolyläthylamins in der Geburtshilfe. Zentralblatt für Gynäkologie 8, 265–269.

[B49] Jones B. L. Kearns G. L. (2011). Histamine: New Thoughts about a Familiar Mediator. Clin. Pharmacol. Ther. 89 (2), 189–197. 10.1038/clpt.2010.256 21178984

[B50] Jutel M. Akdis M. Akdis C. A. (2009). Histamine, Histamine Receptors and Their Role in Immune Pathology. Clin. Exp. Allergy 39 (12), 1786–1800. 10.1111/j.1365-2222.2009.03374.x 20085595

[B51] Kirchhefer U. Baba H. A. Hanske G. Jones L. R. Kirchhof P. Schmitz W. (2004). Age-dependent Biochemical and Contractile Properties in Atrium of Transgenic Mice Overexpressing Junctin. Am. J. Physiology-Heart Circulatory Physiol. 287 (5), H2216–H2225. 10.1152/ajpheart.00137.2004 15205169

[B52] Klocker J. Mätzler S. A. Huetz G.-N. Drasche A. Kolbitsch C. Schwelberger H. G. (2005). 4. Synthesis, Metabolism and Release of Histamine. Inflamm. Res. 54 (Suppl. 1), S54–S57. 10.1007/s00011-004-0425-7 15928834

[B53] Laemmli U. K. (1970). Cleavage of Structural Proteins during the Assembly of the Head of Bacteriophage T4. Nature 227, 680–685. 10.1038/227680a0 5432063

[B54] Layritz C. M. Hagel A. F. Graf V. Reiser C. Klinghammer L. Ropers D. (2014). Histamine in Atrial Fibrillation (AF) - Is There Any Connection? Results from an Unselected Population. Int. J. Cardiol. 172 (3), e432–e433. 10.1016/j.ijcard.2013.12.185 24476699

[B55] Levi R. Malm J. R. Bowman F. O. Rosen M. R. (1981). The Arrhythmogenic Actions of Histamine on Human Atrial Fibers. Circ. Res. 49 (2), 545–550. 10.1161/01.res.49.2.545 7249288

[B56] Livak K. J. Schmittgen T. D. (2001). Analysis of Relative Gene Expression Data Using Real-Time Quantitative PCR and the 2−ΔΔCT Method. Methods 25 (4), 402–408. 10.1006/meth.2001.1262 11846609

[B57] Lowry O. Rosebrough N. Farr A. L. Randall R. (1951). Protein Measurement with the Folin Phenol Reagent. J. Biol. Chem. 193 (1), 265–275. 10.1016/s0021-9258(19)52451-6 14907713

[B58] Maintz L. Bieber T. Novak N. (2006). Die verschiedenen Gesicher der Histaminintoleranz. Deutsches Ärzteblatt 103 (51-52), B3027–B3033.

[B59] Matsuda N. Hattori Y. Sakuraya F. Kobayashi M. Zhang X.-H. Kemmotsu O. (2002). Hemodynamic Significance of Histamine Synthesis and Histamine H 1 - and H 2 -receptor Gene Expression during Endotoxemia. Naunyn-Schmiedeberg’s Arch. Pharmacol. 366 (6), 513–521. 10.1007/s00210-002-0651-x 12444491

[B60] Matsuda N. Jesmin S. Takahashi Y. Hatta E. Kobayashi M. Matsuyama K. (2004). Histamine H1 and H2 Receptor Gene and Protein Levels Are Differentially Expressed in the Hearts of Rodents and Humans. J. Pharmacol. Exp. Ther. 309 (2), 786–795. 10.1124/jpet.103.063065 14752062

[B61] Meister J. Weisgut J. Gergs U. Neumann J. (2015). Human H_2_ Receptors in a Mouse Model and the Endogenous Cardiac Histamine Content. Acta Physiol. 215, S705.

[B62] National Research Council (2011). Guide for the Care and Use of Laboratory Animals. Eighth Edition. Washington, DC: The National Academies Press. 10.17226/12910

[B63] Neumann J. Boknik P. Matherne G. P. Lankford A. Schmitz W. (2003). Pertussis Toxin Sensitive and Insensitive Effects of Adenosine and Carbachol in Murine Atria Overexpressing A1 -adenosine Receptors. Br. J. Pharmacol. 138 (1), 209–217. 10.1038/sj.bjp.0705012 12522092 PMC1573638

[B64] Ogasawara M. Yamauchi K. Satoh Y.-i. Yamaji R. Inui K. Jonker J. W. (2006). Recent Advances in Molecular Pharmacology of the Histamine Systems: Organic Cation Transporters as a Histamine Transporter and Histamine Metabolism. J. Pharmacol. Sci. 101 (1), 24–30. 10.1254/jphs.fmj06001x6 16648665

[B65] Ohtsu H. Tanaka S. Terui T. Hori Y. Makabe-Kobayashi Y. Pejler G. (2001). Mice Lacking Histidine Decarboxylase Exhibit Abnormal Mast Cells. FEBS Lett. 502 (1-2), 53–56. 10.1016/s0014-5793(01)02663-1 11478947

[B66] Panula P. Chazot P. L. Cowart M. Gutzmer R. Leurs R. Liu W. L. S. (2015). International Union of Basic and Clinical Pharmacology. XCVIII. Histamine Receptors. Pharmacol. Rev. 67 (3), 601–655. 10.1124/pr.114.010249 26084539 PMC4485016

[B68] Patella V. Marinò I. Lampärter B. Arbustini E. Adt M. Marone G. (1995). Human Heart Mast Cells. Isolation, Purification, Ultrastructure, and Immunologic Characterization. J. Immunol. 154 (6), 2855–2865. 7533185

[B69] Patella V. Marinò I. Arbustini E. Lamparter-Schummert B. Verga L. Adt M. (1998). Stem Cell Factor in Mast Cells and Increased Mast Cell Density in Idiopathic and Ischemic Cardiomyopathy. Circulation 97 (10), 971–978. 10.1161/01.cir.97.10.971 9529265

[B70] Petch M. C. Nayler W. G. (1979). Concentration of Catecholamines in Human Cardiac Muscle. Heart 41 (3), 340–344. 10.1136/hrt.41.3.340 PMC482037 426982

[B71] Pönicke K. Gergs U. Buchwalow I. B. Hauptmann S. Neumann J. (2012). On the Presence of Serotonin in Mammalian Cardiomyocytes. Mol. Cel. Biochem. 365 (1-2), 301–312. 10.1007/s11010-012-1270-6 22367115

[B72] Sakuma I. Gross S. S. Levi R. (1988). Positive Inotropic Effect of Histamine on guinea Pig Left Atrium: H1-Receptor-Induced Stimulation of Phosphoinositide Turnover. J. Pharmacol. Exp. Ther. 247 (2), 466–472. 2846821

[B73] Sanders L. Lynham J. A. Kaumann A. J. (1996). Chronic ?1-adrenoceptor Blockade Sensitises the H1 and H2 Receptor Systems in Human Atrium: Role of Cyclic Nucleotides. Naunyn-schmiedeberg's Arch. Pharmacol. 353 (6), 661–670. 10.1007/bf00167185 8738299

[B74] Sattler J. Häfner D. Klotter H. J. Lorenz W. Wagner P. K. (1988). Food-induced Histaminosis as an Epidemiological Problem: Plasma Histamine Elevation and Haemodynamic Alterations after Oral Histamine Administration and Blockade of Diamine Oxidase (DAO). Agents Actions 23 (3-4), 361–365. 10.1007/bf02142588 3134804

[B75] Sattler J. Hesterberg R. Lorenz W. Schmidt U. Crombach M. Stahlknecht C. D. (1985). Inhibition of Human and Canine Diamine Oxidase by Drugs Used in an Intensive Care Unit: Relevance for Clinical Side Effects? Agents Actions 16 (3-4), 91–94. 10.1007/bf01983109 3925736

[B76] Saura J. Kettler R. Da Prada M. Richards J. (1992). Quantitative Enzyme Radioautography with 3H-Ro 41-1049 and 3H-Ro 19- 6327 In Vitro: Localization and Abundance of MAO-A and MAO-B in Rat CNS, Peripheral Organs, and Human Brain. J. Neurosci. 12 (5), 1977–1999. 10.1523/jneurosci.12-05-01977.1992 1578281 PMC6575899

[B77] Schenk P. (1921). Uber die Wirkungsweise des β-Imidazolyläthylamins (Histamin) auf den menschlichen Organismus. Archiv F. Experiment. Pathol. U. Pharmakol 89, 332–339. 10.1007/bf01998687

[B78] Schwelberger H. G. Feurle J. Houen G. (2017). Monoclonal Antibodies for Human and Porcine Histamine N-Methyltransferase (HMT) Facilitate Protein Expression and Localization Studies. Inflamm. Res. 66, 67–77. 10.1007/s00011-016-0987-1 27632021 PMC5209425

[B79] Schwelberger H. G. Feurle J. Houen G. (2013). New Tools for Studying Old Questions: Antibodies for Human Diamine Oxidase. J. Neural Transm. 120, 1019–1026. 10.1007/s00702-012-0936-2 23238973

[B80] Schwelberger H. G. (2010). Structural Organization of Mammalian Copper-Containing Amine Oxidase Genes. Inflamm. Res. 59 (Suppl. 2), S223–S225. 10.1007/s00011-009-0135-2 20013028

[B81] Seifert R. Strasser A. Schneider E. H. Neumann D. Dove S. Buschauer A. (2013). Molecular and Cellular Analysis of Human Histamine Receptor Subtypes. Trends Pharmacol. Sci. 34 (1), 33–58. 10.1016/j.tips.2012.11.001 23254267 PMC3869951

[B82] Singh S. Johnson P. I. Javed A. Gray T. S. Lonchyna V. A. Wurster R. D. (1999). Monoamine- and Histamine-Synthesizing Enzymes and Neurotransmitters Within Neurons of Adult Human Cardiac Ganglia. Circulation 99 (3), 411–419. 10.1161/01.cir.99.3.411 9918529

[B83] Skovgaard N. Møller K. Gesser H. Wang T. (2009). Histamine Induces Postprandial Tachycardia through a Direct Effect on Cardiac H2-Receptors in Pythons. Am. J. Physiology-Regulatory, Integr. Comp. Physiol. 296 (3), R774–R785. 10.1152/ajpregu.90466.2008 19091908

[B84] Smith M. J. Garrett R. H. (2005). A Heretofore Undisclosed Crux of Eosinophilia-Myalgia Syndrome: Compromised Histamine Degradation. Inflamm. Res. 54 (11), 435–450. 10.1007/s00011-005-1380-7 16307217

[B85] Tahara A. Nishibori M. Ohtsuka A. Sawada K. Sakiyama J. Saeki K. (2000). Immunohistochemical Localization of HistamineN-Methyltransferase in Guinea Pig Tissues. J. Histochem. Cytochem. 48 (7), 943–954. 10.1177/002215540004800707 10858271

[B86] Takai J. Ohtsu H. Sato A. Uemura S. Fujimura T. Yamamoto M. (2019). Lipopolysaccharide-induced Expansion of Histidine Decarboxylase-Expressing Ly6G+ Myeloid Cells Identified by Exploiting Histidine Decarboxylase BAC-GFP Transgenic Mice. Sci. Rep. 9 (1), 15603. 10.1038/s41598-019-51716-6 31666556 PMC6821885

[B87] Taylor K. M. Snyder S. H. (1972). Isotopic Microassay of Histamine, Histidine, Histidine Decarboxylase and Histamine Methyltransferase in Brain Tissue. J. Neurochem. 19 (5), 1343–1358. 10.1111/j.1471-4159.1972.tb01459.x 4401996

[B88] Towbin H. Staehelin T. Gordon J. (1979). Electrophoretic Transfer of Proteins from Polyacrylamide Gels to Nitrocellulose Sheets: Procedure and Some Applications. Proc. Natl. Acad. Sci. 76, 4350–4354. 10.1073/pnas.76.9.4350 388439 PMC411572

[B89] von Dorsche H. H. Stiller D. Pambor M. Schwesinger G. Sodemann S. Stolp A. (1981). Nachweis der Naphthol-AS-D-Chloracetat-Esterase-Aktivität in Mastzellen unter besonderer Berücksichtigung cutaner Mastocytosen. Acta Histochem. 69 (1), 23–IN6. 10.1016/s0065-1281(81)80005-0 6795886

[B90] Waldmeier P. C. Feldtrauer J.-J. Maǐtre L. (1977). Methylhistamine: Evidence for Selective Deamination by MAO B in the Rat Brain In Vivo. J. Neurochem. 29 (5), 785–790. 10.1111/j.1471-4159.1977.tb10719.x 591954

[B91] Wantke F. Proud D. Siekierski E. Kagey-Sobotka A. (1998). Daily Variations of Serum Diamine Oxidase and the Influence of H1 and H2 Blockers: a Critical Approach to Routine Diamine Oxidase Assessment. Inflamm. Res. 47 (10), 396–400. 10.1007/s000110050350 9831324

[B92] Weiss S. Robb G. P. Ellics L. B. (1932). The Systemic Effects of Histamine in Man. Arch. Intern. Med. (Chic) 49 (3), 360–396. 10.1001/archinte.1932.00150100017002

[B93] Wiener Z. Tóth S. Gócza E. Kobolák J. Falus A. (2003). Mouse Embryonic Stem Cells Express Histidine Decarboxylase and Histamine H1 Receptors. Inflamm. Res. 52 (Suppl. 1), S53–S54. 10.1007/s000110300052 12755409

[B94] Windaus A. Vogt W. (1907). Synthese des Imidazolyl-äthylamins. Ber. Dtsch. Chem. Ges. 40, 3691–3695. 10.1002/cber.190704003164

[B95] Wolff A. A. Levi R. (1986). Histamine and Cardiac Arrhythmias. Circ. Res. 58 (1), 1–16. 10.1161/01.res.58.1.1 2417741

[B96] Wollin A. Wang X. Tso P. (1998). Nutrients Regulate Diamine Oxidase Release from Intestinal Mucosa. Am. J. Physiology-Regulatory, Integr. Comp. Physiol. 275 (4 Pt 2), R969–R975. 10.1152/ajpregu.1998.275.4.r969 9756524

[B97] Yoshikawa T. Naganuma F. Iida T. Nakamura T. Harada R. Mohsen A. S. (2013). Molecular Mechanism of Histamine Clearance by Primary Human Astrocytes. Glia 61 (6), 905–916. 10.1002/glia.22484 23505051

[B98] Zerkowski H.-R. Broede A. Kunde K. Hillemann S. Schäfer E. Vogelsang M. (1993). Comparison of the Positive Inotropic Effects of Serotonin, Histamine, Angiotensin II, Endothelin and Isoprenaline in the Isolated Human Right Atrium. Naunyn-schmiedeberg's Arch. Pharmacol. 347 (4), 347–352. 10.1007/bf00165383 8389986

[B99] Zimmermann A. S. Burhenne H. Kaever V. Seifert R. Neumann D. (2011). Systematic Analysis of Histamine and N-Methylhistamine Concentrations in Organs from Two Common Laboratory Mouse Strains: C57Bl/6 and Balb/c. Inflamm. Res. 60 (12), 1153–1159. 10.1007/s00011-011-0379-5 21912978

