# Supplementary material for: Histamine can be Formed and Degraded in the Human and Mouse Heart
Source: Front Pharmacol. 2021 May 11;12:582916. doi: 10.3389/fphar.2021.582916 (PMC8144513; doi:10.3389/fphar.2021.582916)
Supplement: Supplementary file 1 [file Image2.PDF]

Supplementary Figure 2

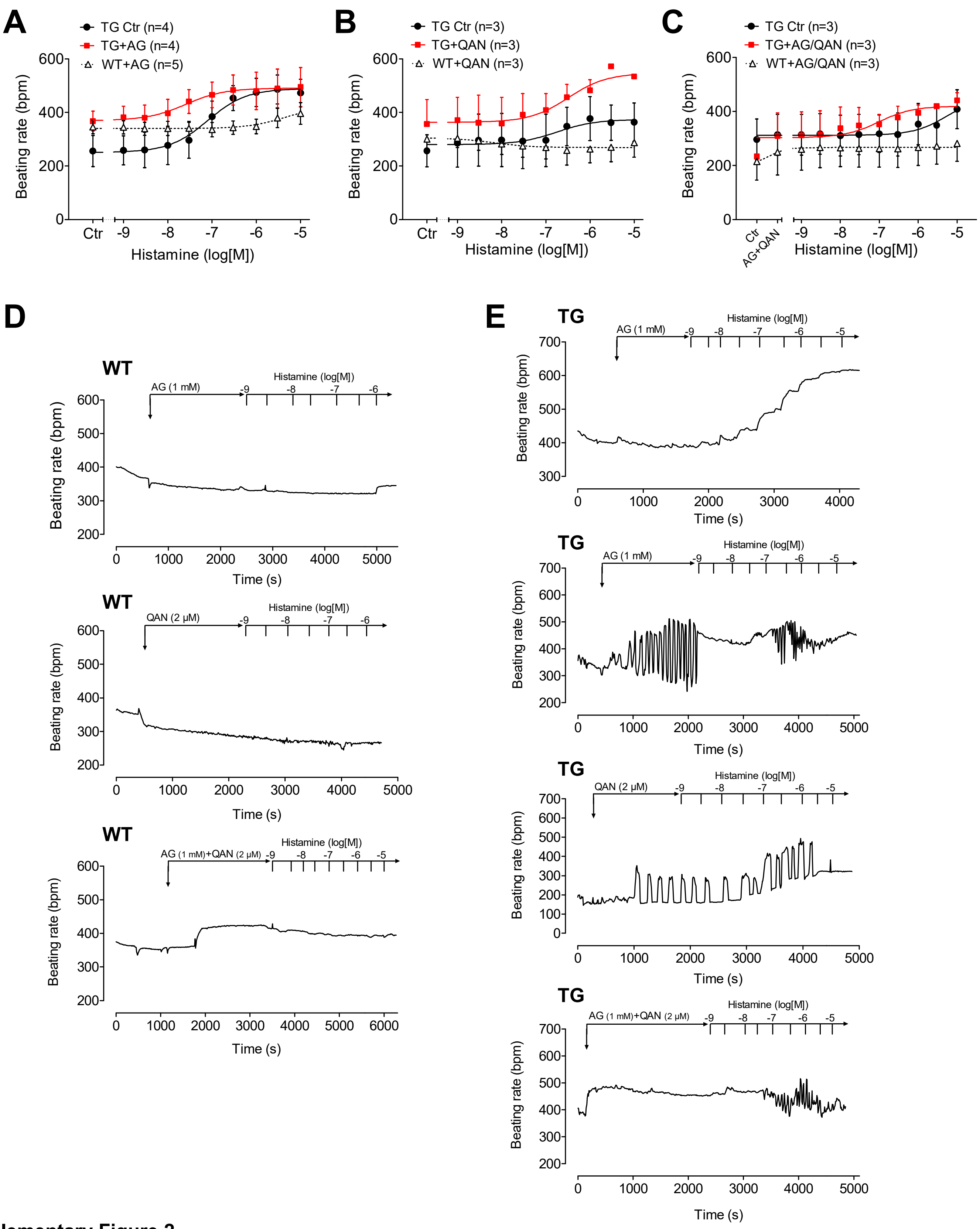

Supplementary Figure 2

Effects of inhibitors of histamine metabolizing enzymes on beating rate of isolated right atrium of H_2-receptor overexpressing mice (TG) or wild-type controls (WT). (**A**) Effect of histamine alone (TG Ctr) or in the presence of 1 mM aminoguanidine (TG+AG). (**B**) Effect of histamine alone (TG Ctr) or in the presence of 2 µM quinacrine (TG+QAN). (**C**) Effect of histamine alone (TG Ctr) or in the combined presence of 1 mM aminoguanidine and 2 µM quinacrine (TG+AG/QAN). WT preparations (WT+AG; WT+QAN; WT+AG/QAN) are shown to demonstrate the generally missing histamine effect in WT. (**D, E**) Original recordings of inhibitors and concentration-response-curves of histamine on the beating rate of isolated right atrial preparations from WT (**D**) and H_2-TG (**E**) mice. Note the high incidence of arrhythmias in TG right atrial preparations, making it difficult to properly analyze any effects of the inhibitors. Ctr, control value before drug addition.
