# Supplementary material for: Histamine can be Formed and Degraded in the Human and Mouse Heart
Source: Front Pharmacol. 2021 May 11;12:582916. doi: 10.3389/fphar.2021.582916 (PMC8144513; doi:10.3389/fphar.2021.582916)
Supplement: Supplementary file 2 [file Image1.PDF]

Supplementary Figure 1

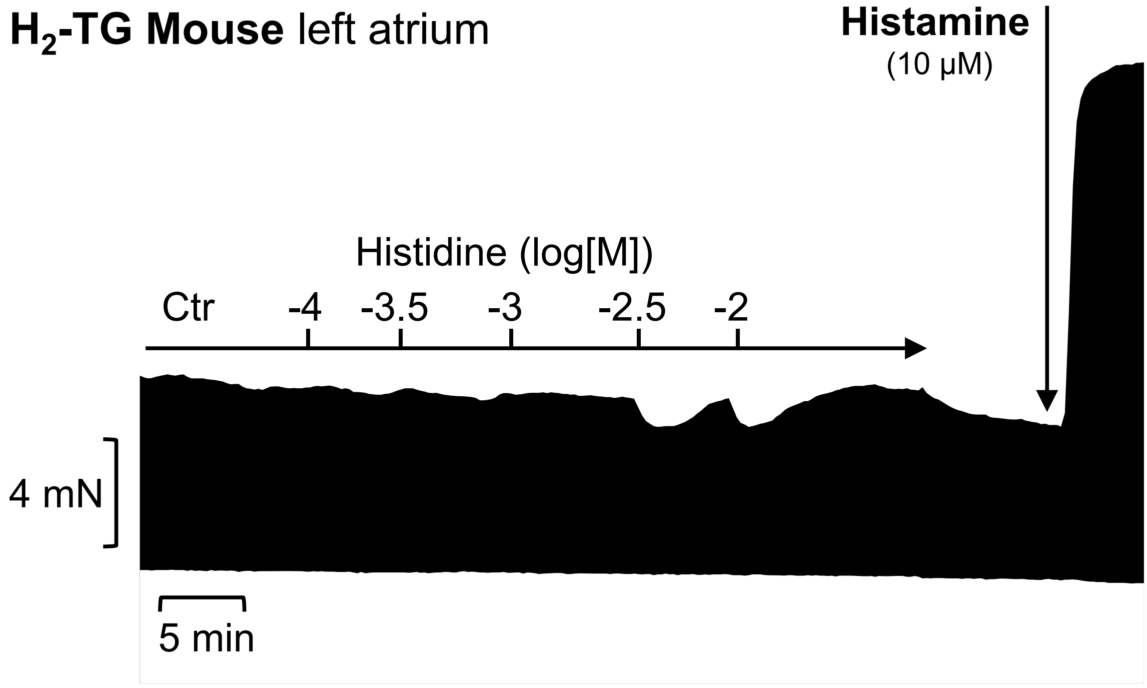

Supplementary Figure 1

Representative original recording of the concentration- and time-dependent effect of histidine on the force of contraction in isolated electrically driven (1 Hz) left atrial preparation from H_2-TG mouse. As positive control, a high concentration (10 µM) of histamine was added at the end of the experiment. Ctr, control value before drug addition.
